# Cashew Gum/Pure and Modified Hydroxyapatite Nanocomposite Aerogels for Water-Pollutant Removal

**DOI:** 10.3390/polym18182306

**Published:** 2026-09-20

**Authors:** Joyce Araujo Borges, Ariane Maria da Silva Santos, Erica Ianne da Silva Sousa, Edvani Curti Muniz, Josy Antevelli Osajima, Luís Humberto de Oliveira, Maria Gardênnia Fonseca, Rafael de Carvalho Araújo, Edson C. Silva-Filho

**Affiliations:** 1 LIMAV—Interdisciplinary Laboratory for Advanced Materials, Campus Ministro Petrônio Portella, Federal University of Piauí, Teresina 64049-550, Brazil; joyceab@ufpi.edu.br (J.A.B.); ariane.am42@gmail.com (A.M.d.S.S.); ericaianne123@ufpi.edu.br (E.I.d.S.S.); curtimuniz@gmail.com (E.C.M.); josyosajima@ufpi.edu.br (J.A.O.); luishumbertodeoliveira@gmail.com (L.H.d.O.); 2 Research and Extension Center-Fuel and Materials Laboratory (NPE–LACOM), Federal University of Paraíba—UFPB, João Pessoa 58059-900, Brazil; mgardennia@gmail.com (M.G.F.); rafaell.ufpb@gmail.com (R.d.C.A.)

**Keywords:** aerogel, cashew gum, hydroxyapatite, adsorption, methylene blue

## Abstract

The pollution of aquatic environments by synthetic dyes, especially those from the textile industry, represents a significant environmental challenge. In this work, nanocomposite aerogels based on cashew gum (CG) and pure and aminosilane-modified hydroxyapatite (HAp) were synthesized and characterized for their application in the adsorption of the methylene blue dye. X-ray diffraction (XRD), Fourier-transform infrared spectroscopy (FTIR), CHN elemental analysis, thermogravimetric analysis (TGA), and scanning electron microscopy (SEM) were used to characterize the materials. Characterization results confirmed the incorporation of HAp into the polymer matrix and its modification, as well as changes in the morphological and thermal properties of the aerogels. The materials exhibited swelling capacity, with a higher degree of swelling observed in basic media. Regarding methylene blue adsorption, equilibrium was reached after 180 min, with varying adsorption capacities among the aerogels. The highest adsorption capacity was observed at pH 12, and kinetic data was best described by the pseudo-first-order model. Equilibrium data showed the best fit to the Langmuir and Dubinin–Radushkevich models, depending on the material. The temperature had no significant effect on the amount of dye adsorbed by the aerogels in the 25 to 55 °C range. The results demonstrate that combining cashew gum with hydroxyapatite, alongside modification of the mineral phase, enables the production of aerogels with tunable structural and adsorptive properties, highlighting their potential for removing cationic dyes from aqueous media.

## 1. Introduction

The indiscriminate dumping of organic pollutants into the environment without prior treatment is a major cause of aquifer and soil contamination, compromising water quality, marine life, and soil fertility [1]. Synthetic dyes are organic moieties widely used in the textile industry because they provide more intense and vibrant colors, making them one of the main water pollutants. They are difficult to decompose due to their physical-chemical and thermal stability [2,3], and are often disposed of incorrectly [4].

Methylene blue, also called tetramethylthioninium chloride or methylene blue chloride, 3,7-bis(dimethylamino) phenothiazine chloride, is a non-biodegradable cationic synthetic dye, with textile applications, being used to color wool, paper, silk, cotton [5,6]. It is a toxic, mutagenic, and carcinogenic pollutant that can cause problems in the human respiratory tract, eye burns, and, if ingested, can cause nausea, burning, and vomiting [1,5,7]. In the aquatic environment, its molecules can prevent sunlight from penetrating, hindering photosynthesis and causing ecosystem degradation [8].

The environmental impact and the growing concern about the scarcity and contamination of water resources, global initiatives have been strengthened to promote the sustainable use of water. The UN 2030 Agenda establishes the Sustainable Development Goals (SDGs) 6.1 and 6.3 of the United Nations, which refer to the importance of ensuring universal access to clean and safe water, as well as promoting the treatment and recovery of wastewater, improving its quality by reducing pollution, eliminating inadequate discharges, and minimizing the release of hazardous chemicals and materials.

Many methodologies are employed for wastewater decontamination, among which adsorption [9] stands out by allowing pollutant molecules to bind to the adsorbent surface through various interactions and interactions-types. Furthermore, it offers advantages such as operational simplicity, safety, high efficiency, and the possibility of adsorbent regeneration and reuse, making its large-scale application economically viable [10,11,12]. Activated carbon [13], carbon nanotubes [14], clays [15], and polymeric materials [16] are commonly used in adsorption processes. Among the polymeric materials used in adsorption, polysaccharides stand out, as they can be crosslinked to form hydrogels that have proven to be a good alternative for this application. Polysaccharides are biodegradable, non-toxic, and economically viable, and they exhibit good adsorption capacity due to the diversity of functional groups in their structures [1,11,17].

Hydrogels are three-dimensional networks of polymers that, due to their hydrophilic groups, can retain large amounts of solutes [12,18]. They can be produced from synthetic polymers, such as polyacrylamide (PAM) and poly(ethylene glycol) (PEG), as well as from natural polymers, such as polysaccharides and gums [18]. Natural gums are polysaccharides that can hydrate when in contact with water and, due to the branching in their structure, exhibit cohesive and adhesive properties [19,20]. In addition, they can be obtained from exudates [18], algae [21], microbial fermentation [22], etc.

Among the gums, we can highlight cashew gum, obtained from the exudate of *Anacardium occidentale* Linn (family Anacardiaceae), a species widely distributed in northeastern Brazil [19,23]. Cashew gum is biodegradable and biocompatible and can be used in drug delivery systems, agriculture, and the cosmetic and food industries [20,24]. Cashew gum (CG) is an anionic heteropolysaccharide that exhibits a branched structure composed of galactose (72–73%), glucose (11–14%), glucuronic acid (4.7–6.3%), arabinose (4.6–5%), rhamnose (3.2–4%), and mannose (1–2%) units. Its chain contains β(1 → 3) and β(1 → 6) linkages, as well as hydroxyl (–OH) and carboxyl groups, which confer hydrophilic properties and facilitate interactions with molecules in solution [25,26]. Furthermore, the composition of GC can vary depending on its origin, exudation time, etc. Thus, the monosaccharide composition, the degree of branching, and the presence of ionizable groups collectively contribute to the physicochemical and functional properties of cashew gum. Cashew gum has been used as a substitute for Arabic gum, reducing production costs and serving as an economically viable alternative in the Brazilian Northeast, as it does not require importation [18,23,27]. Therefore, due to its ease of availability and abundance in that region, cashew gum enables the production of biodegradable hydrogels from a completely natural and renewable source [28].

Some studies have been conducted in this direction, including that of Silva et al. [29], who developed a superabsorbent nanocomposite aerogel from cashew gum with laponite and applied it to the adsorption of the cationic dye crystal violet. The results indicated that the insertion of laponite into the aerogel not only improved the adsorption kinetics and the maximum removal capacity but also conferred selectivity for cationic dyes in mixtures. Therefore, the authors concluded that HCG-LAP is a promising, efficient adsorbent suitable for water treatment, with low toxicity and good reusability.

Furthermore, this gum can interact with inorganic matrices, resulting in composites with greater chemical stability. Among these matrices, we can mention hydroxyapatite (Ca_10_(PO_4_)_6_(OH)_2_), a calcium phosphate-based biomaterial that has chemical similarity to bones and teeth [28] and can be found in nature or obtained synthetically. Because it is poorly soluble in water, has a high ion-exchange capacity, and can adsorb both anionic and cationic species, it has been used to remove pollutants from wastewater with successful results [3,7]. Thus, combining cashew gum and hydroxyapatite to form a nanocomposite, for example, can enhance properties or generate a synergistic effect that favors adsorption. The gum provides a hydrophilic matrix rich in oxygen-containing groups and possesses a high swelling capacity, which facilitates hydration [30]. Hydroxyapatite, in turn, contributes surface groups, such as phosphates, capable of interacting with dyes [31]. The combination of these two phases can, therefore, modify the material’s surface and adsorption properties.

Aaddouz et al. [7] synthesized hydroxyapatite nanoparticles using two different methods and evaluated their application in methylene blue removal. The materials exhibited a maximum adsorption capacity of 40 mg g^−1^, reaching equilibrium within 20 min, with adsorption favored under basic pH conditions at 25 °C. The adsorption kinetics followed the pseudo-second-order model, while the Freundlich isotherm indicated multilayer adsorption. Allam et al. [32] investigated methylene blue removal using microwave-irradiated hydroxyapatite, achieving equilibrium within 4 min and a maximum adsorption capacity of 33.3 mg g^−1^, considerably higher than that of non-irradiated HAp (0.7 mg g^−1^). Adsorption was favored at pH 12, followed pseudo-second-order kinetics, and was better fitted by the Langmuir isotherm, indicating monolayer adsorption. Peighambardoust et al. [33] developed activated carbon (AC), activated carbon/hydroxyapatite (AC/HAp), and carboxymethylcellulose/activated carbon/hydroxyapatite (CMC/AC/HAp) adsorbents for the removal of methylene blue, with adsorption capacities of 40.0, 44.25, and 43.86 mg g^−1^, respectively. The process, carried out at pH 8 for 60 min, followed pseudo-second-order kinetics and the Langmuir isotherm, exhibiting spontaneous and exothermic behavior.

Adsorption by hydroxyapatite can occur via mechanisms such as ion exchange, surface complexation, and electrostatic interactions, thereby enabling it to remove various classes of pollutants [31]. In addition, methods that functionalize and surface-modify hydroxyapatite using coupling agents, such as silanes, can enhance nanoparticle-polymer interactions and improve pollutant adsorption efficiency, thereby allowing greater interaction with the pollutant [34,35]. Modifying hydroxyapatite with agents such as aminosilanes, for example, 3-aminopropyltrimethoxysilane, introduces amino groups onto its surface, thereby altering surface interaction characteristics [36]. These functional groups can enhance interactions with the organic phase, contributing to improved integration between the inorganic and polymeric phases [37], and the presence of amino groups can increase the availability of sites capable of interacting with chemical species present in the medium. Although cashew gum and hydroxyapatite show potential for adsorption applications, their combination in aerogels, particularly with modified hydroxyapatite, remains underexplored. The interaction between the phases can be influenced by surface charge and the availability of functional groups. Therefore, this study aimed to develop new nanocomposite aerogels based on cashew gum and hydroxyapatite, either pure or modified with 3-aminopropyltrimethoxysilane, 3-propylethylenediaminetrimethoxysilane, or 3-propyldiethylenetriaminotrimethoxysilane, for the adsorption of the dye methylene blue. Methylene blue was selected as a model molecule due to its relevance as a textile industry contaminant and its widespread use in adsorption studies. The materials were characterized regarding their chemical, structural, thermal, and morphological properties, and subsequently evaluated for swelling behavior and dye adsorption capacity, including an assessment of kinetic and equilibrium parameters.

## 2. Materials and Methods

### 2.1. Materials

3-aminopropyltrimethoxysilane (97.0%), 3-propylethylenediaminotrimethoxysilane (97.0%), 3-propyldiethyltriaminotrimethoxysilane (97.0%), Acrylamide (98.0%); N, N,N′,N′–tetramethylenediamine—TEMED (99.0%), Potassium persulfate—KPS (99.0%), N,N′-methylenebisacrylamide—MBA (99.0%), Sodium hydroxide—NaOH (97.0%) and Potassium bicarbonate—KHCO_3_ (99.5%) were provided by Sigma-Aldrich (St. Louis, MO, USA). Methanol—CH_3_OH (99.8%), Ethanol—C_2_H_5_OH (99.8%); Methylene Blue dye—C_16_H_18_N_3_SCl and Calcium hydroxide—Ca(OH)_2_ (95.0%) was provided by Dinâmica, São Paulo, Brazil. Dibasic ammonium phosphate—(NH_4_)_2_HPO_4_ (98.0%), Hydrochloric acid—HCl (37.0%) and Hydrochloric acid—HCl (37.0%) were provided by Neon, São Paulo, Brazil. Toluene—C_6_H_5_CH_3_ (99.5) was provided by Impex (Santos, Brazil). Acetone—C_3_H_6_O was from Greentech, São Paulo, Brazil. The cashew gum exudate was collected from trees at the Federal University of Piauí—UFPI (SISGEN: ABD61DA).

### 2.2. Synthesis and Modification of Hydroxyapatite

The synthesis of hydroxyapatite was carried out according to a previous study [26], with some adaptations. For the synthesis of 10.0 g of hydroxyapatite, a solution was initially prepared by dissolving 7.37 g of Ca(OH)_2_ in 250 mL of distilled water and 7.88 g of (NH_4_)_2_HPO_4_ in 250 mL of distilled water. The solutions were stirred separately so that the reagents dissolved completely in the water. After this step, the Ca(OH)_2_ solution was slowly added to the (NH_4_)_2_HPO_4_ solution, and the mixture was stirred for 4 h at 25 ± 2 °C. The solution was then left to stand for 12 h. The white precipitate formed was then centrifuged, washed with distilled water, and dried in an oven at 100 °C for 24 h. The solid was named HAp. The modification of HAp was carried out as described in a previous study [3], with some modifications. Initially, 5.0 g of obtained HAp was dispersed in 100 mL of toluene at 100 °C under a nitrogen atmosphere with magnetic stirring. Then, 10 mL of the silylating agent 3-aminopropyltrimethoxysilane was added. The system was stirred for 96 h at 110 °C. After this period, the system was cooled, and the product obtained was washed with toluene, ethanol, and water, and dried in an oven at 80 °C for 24 h. The HAp modified with 3-aminopropyltrimethoxysilane was named HApN1. The modification of HAp with the silanes 3-propylethylenediaminotrimethoxysilane and 3-propyldiethylenetriaminotrimethoxysilane occurred under the same conditions described above, and the products obtained were named HApN2 and HApN3, respectively. The structures of silanes are illustrated in Figure S1 of the Supplementary Materials.

### 2.3. Obtaining Cashew Gum

Cashew gum was obtained after isolating the exudate found in fissures present in cashew trees (*Anacardium occidentale* L.). The collected exudate was manually processed to remove impurities, such as encrusted branches and leaves. After this step, the exudate was dispersed in distilled water at a ratio of 10.0 g per 100 mL, and the mixture was mechanically agitated (400 rpm) for 24 h at 25 °C. The dispersion was then filtered and the pH adjusted to 7 using a solution of HCl and/or NaOH (1.0 mol L^−1^). Subsequently, ethanol was added to the solution in a 3:1 (*v*/*v*) ratio. The precipitate was then centrifuged at 5000 rpm, washed three times with ethanol and once with acetone, and dried in an oven at 50 °C [18,26]. Figure S2 illustrates images of (a) cashew tree exudate and (b) cashew tree gum obtained after purification of the precursor material.

### 2.4. Synthesis of Aerogels

The synthesis of aerogels was carried out according to previous studies by de Lima et al. [38], with some modifications. Initially, 3.0 g of cashew gum were dispersed in 45 mL of distilled water under magnetic stirring. Then, 2.1 g of acrylamide, 0.016 g of potassium persulfate, 0.024 g of bisacrylamide, 0.1 g of potassium bicarbonate, and 100 µL of TEMED were added to the system. The synthesis was carried out under a N_2_(g) atmosphere for 24 h. The obtained material was then washed with methanol (30% *v*/*v*) and dried in an oven at 70 °C for 24 h. After drying, 1.0 g of the dried material was added to 40 mL of NaOH solution (0.5 mol L^−1^). The system was maintained under magnetic stirring at 50 °C for 1 h. The material was then washed with distilled water, frozen, and subsequently lyophilized. The aerogel was named HCG. The synthesis of cashew gum nanocomposite aerogels containing pure and modified hydroxyapatite followed the same methodology as for HCG; however, in the gum dissolution step, 20% by mass of HAp was added to the medium relative to the gum and acrylamide, and for the other systems, 20% by mass of HapN1, HapN2, and HapN3 were added. The systems were agitated until the hydroxyapatite was completely dispersed in the mixture. The aerogels obtained after the other steps were named HHAp, HHApN1, HHApN2, and HHApN3.

### 2.5. Characterizations

The materials were subjected to X-ray diffraction analysis using a D8 Advance diffractometer (Bruker AXS, Karlsruhe, Germany) with CuKα radiation (λ = 1.5418 Å) at 40 kV/40 mA. All analyses were performed over the 2θ range from 5 to 90°, with a step size of 0.01° and an acquisition time of 1 s/step.

Fourier transform infrared (FTIR) spectra of the precursors, as well as of the cashew gum/hydroxyapatite nanocomposite aerogels with and without chemical modification, were obtained using a Perkin Elmer Spectrum 100 spectrometer (Perkin Elmer, Shelton, CT, USA) in transmittance mode using potassium bromide (KBr) pellets, with 16 scans in the wavenumber range of 4000 to 400 cm^−1^ and a resolution of 4 cm^−1^.

Carbon, hydrogen, and nitrogen content were determined by elemental analysis using a PE 2400 Series II elemental analyzer (Perkin Elmer). The analyses were performed in triplicate, and the results were averaged.

The thermal analysis (TG) measurements derived from the thermal analysis (DTG) of the aerogels were performed on an SDT Q600 instrument (TA Instruments, New Castle, DE, USA). The samples (approximately 5 mg) were accurately weighed into an alumina container and heated from 25 to 600 °C at 10 °C/min under an argon atmosphere at a flow rate of 50 mL min^−1^.

The morphology of the materials was observed using a scanning electron microscope (Quanta FEG 250, FEI, Dreieich, Germany) with an acceleration voltage of 1–30 kV, equipped with an SDD (Silicon drift detector) and a Bruker Quantax EDS model XFlash 5010 detector (Berlim, Germany).

### 2.6. Swelling Capacity

The maximum water-absorption capacity of cashew gum nanocomposite hydrogels was evaluated according to a previous study [18]. Initially, 10.0 mg of dry hydrogels were immersed in 20.0 mL of distilled water at 25 °C. After predetermined times of 15, 30, 60, 90, 120, 150, 180, 210, and 240 min, the swollen hydrogels were removed from the medium, and excess water was removed using a sieve. The swelling ratio was determined by Equation (1):

(1) $$\mathrm{Swelling}\;(\%)=\frac{Mf-Mi}{Mi}\times 100$$

where *Mi* and *Mf* correspond to the masses before and after swelling, respectively.

Swelling tests for the hydrogels were also performed by evaluating water absorption at pHs 2, 4, 7, 10, and 12, with distilled water pH adjusted using HCl (1.0 mol L^−1^) and/or NaOH (1.0 mol L^−1^). The swelling test at different pHs accounted for the hydrogels’ equilibration time and followed the same methodology described above. The entire water absorption capacity study was performed in triplicate.

### 2.7. Point of Zero Charge (pHpcz)

To determine the point of zero charge of the materials, a 0.1 mol L^−1^ NaCl solution was prepared, placed in beakers, and the pH of the solutions was adjusted to 2, 4, 7, 10, and 12, designated as the initial pH (pHi), using 1.0 mol L^−1^ HCl and/or 1.0 mol L^−1^ NaOH. Then, 30.0 mL of each solution with the adjusted pH was transferred to Erlenmeyer flasks containing approximately 10 mg of each material, and the mixture was agitated for 24 h at 25 ± 2 °C. Measurements were taken using an Instrutherm PH-5000 pH meter (São Paulo, Brazil). After this process, the supernatants were centrifuged at 5000 rpm for 3 min, and the pH was measured to determine the final pH (pHf). The change in the point of zero charge, ΔpHpcz, is determined by the difference between the initial and final pH, as shown in Equation (2):

ΔpHpcz = pH_i_ − pH_f_ (2)

The test was performed in triplicate for all aerogels.

### 2.8. Methylene Blue Adsorption Test

The dye adsorption assays were performed according to the methodology described in the literature [29], in which the effects of contact time, pH, and initial dye concentration were evaluated. The tests were performed under agitation, starting from the dispersion of 10.0 mg of each aerogel in 30.0 mL of methylene blue dye solution. All tests were performed in triplicate. After each assay, the aerogels were centrifuged, and aliquots of the supernatant were removed for determination of the dye concentration by UV-Vis spectrophotometry, using a Cary 60 UV-Vis spectrophotometer (Agilent Technologies, Santa Clara, CA, USA), at the maximum absorption wavelength for methylene blue dye, λmax = 665 nm, in accordance with previous studies [39]. The absorption spectrum of methylene blue is illustrated in Figure S3**.** The quantities of dyes adsorbed (q) were calculated according to Equation (3):

(3) $$q=\frac{\left(Ci-Ce\right)\times V}{m}$$

where Ci and Ce are the initial and equilibrium dye concentrations (mg L^−1^), respectively; V is the volume of the dye solution (L), and m is the mass of the aerogel (g). For the evaluation of the kinetic study and pH, the tests were performed using a methylene blue solution at 500 mg L^−1^. The kinetic study was carried out over the time interval 5–720 min at 25 °C and with constant stirring at 110 rpm. In the evaluation of the pH effect, the equilibrium time obtained in the kinetic study was used, varying the pH to 2, 4, 7, 10, and 12, adjusted with 1.0 mol L^−1^ HCl and NaOH solutions. In the effect of dye concentration, initial concentrations of 10–1500 mg L^−1^ (10, 50, 100, 200, 400, 500, 750, 1000, 1250, and 1500 mg L^−1^) of methylene blue dye were used, and the tests were performed under optimal pH and contact time conditions. To evaluate the effect of temperature on dye adsorption, a temperature range of 25, 35, 45 and 55 °C was used, and the tests were conducted under optimal conditions of contact time, pH, and concentration.

### 2.9. Kinetic and Equilibrium Models

The pseudo-first-order [40], second-order [41], and Elovich [42] kinetic models were employed to investigate the adsorption kinetics and are described by Equations (4), (5), and (6), respectively:

ln(q_e_ − q_t_) = ln(q_e_) − k_1_t (4)

(5) $$\frac{t}{q_{t}}=\frac{1}{k_{2}q_{e}^{2}}+\frac{t}{q_{e}}$$

(6) $$q_{t}=\frac{1}{\beta }\;\ln(\alpha \beta )+\frac{1}{\beta }\;\ln(t+t_{o})$$

where qe and qt are the quantities adsorbed in mg g^−1^ at equilibrium and at time t (min). k_1_ (min^−1^) and k_2_ (g mg^−1^ min^−1^) refer, respectively, to the pseudo-first-order and second-order adsorption rate constants, and, for the Elovich model, α (mg g^−1^ min^−1^) and β (mg g^−1^) refer to the initial adsorption rate and desorption constant, respectively.

Adsorption isotherms, in turn, help evaluate and understand the interactions between the adsorbate and adsorbent, as well as determine the maximum adsorption capacity. Based on the results obtained from the equilibrium isotherms, the experimental data were fitted to the Langmuir [43], Freundlich [44], Temkin [45], and Dubinin-Radushkevich (D-R) [46] models, whose representative equations for each model are described below, Equations (7) to (10), respectively:

(7) $$q_{e}=\frac{K_{L}\;q_{max}\;C_{e}}{1+K_{L}\;C_{e}}$$

q_e_ = K_F_C_e_^1/n^ (8)

(9) $$q_{e}=B\ln{(A}_{t}C_{e})$$

q_e_ = q_m_ e^RTln(1+1/Ce)^ (10)

In which qe (mg g^−1^) refers to the amount of solute adsorbed per gram of adsorbent at equilibrium; q_max_ (mg g^−1^) corresponds to the maximum amount of solute adsorbed to form the surface monolayer; C_e_ (mg L^−1^) is the concentration of the adsorbate at equilibrium, and K_L_ (L g^−1^) is the Langmuir constant referring to the interaction between adsorbent and adsorbate. For the Freundlich model, K_F_ and n refer, respectively, to the Freudlich adsorption capacity constant and the adsorption intensity. In the Temkin model, B (J mol^−1^) and At (L mg^−1^) are constants that depend, respectively, on the intensity and adsorption capacity; R is the universal gas constant (8.314 J mol^−1^ K), and T (K) is the temperature. In the Equation describing the D-R isotherm, R is the universal gas constant (8.314 J mol K^−1^), and T is the temperature in Kelvin (K).

## 3. Results and Discussion

### 3.1. X-Ray Diffraction (XRD)

**Figure 1:**
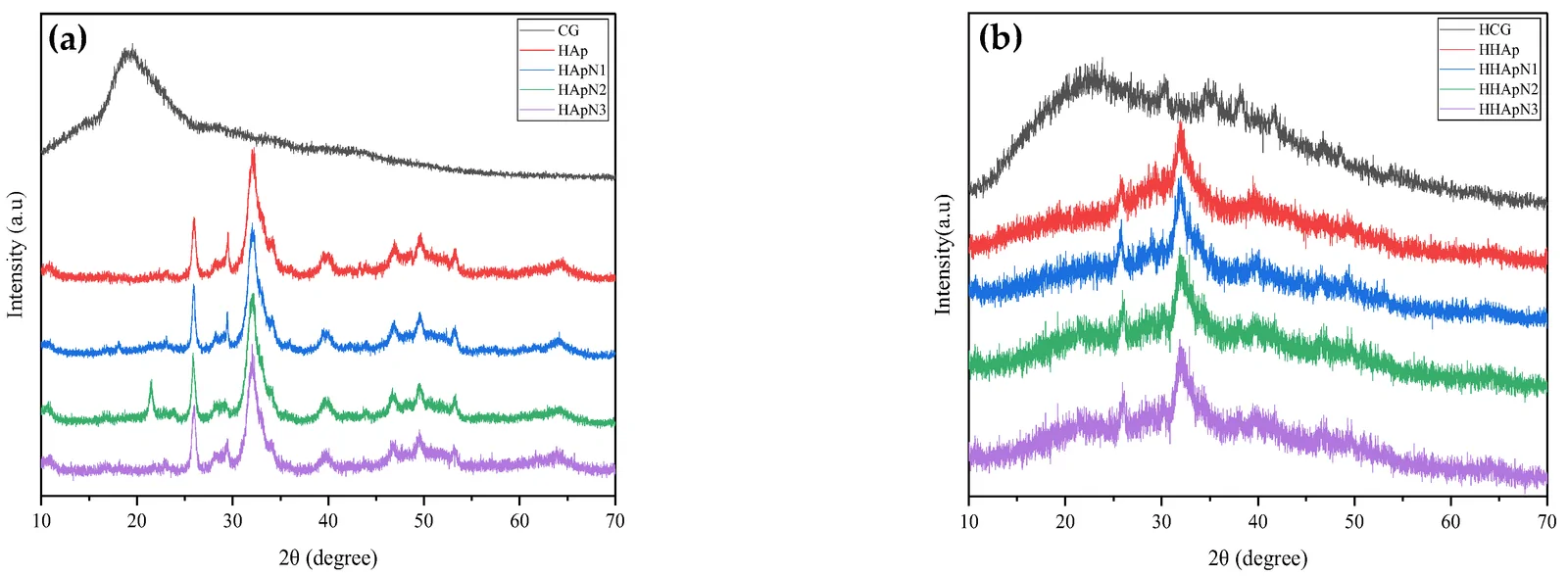
XRD patterns of (**a**) cashew gum, hydroxyapatite, and aminosilane-functionalized hydroxyapatite with different surface functional groups; (**b**) cashew gum aerogels and their composites containing pure and modified hydroxyapatite.

The X-ray diffractograms of the samples before and after aerogel synthesis are shown in Figure 1a and Figure 1b, respectively.

Cashew gum (CG) exhibited an amorphous halo at 2θ ≈ 19.5°, indicating an amorphous structure with a low degree of crystallinity, a common characteristic in polymers and biopolymers [18,26,29]. For pure HAp, refinement was performed using the Rietveld method using the REX 3.9.2 software, in which the main planes of the hydroxyapatite phase were identified according to the ICDD96-900-1234 chart, as illustrated in Figure S4. Reflections with values of 2θ = 25.9°, 32.2°, 39.8°, 46.9°, 49.4° and 53.1° were indexed to the hydroxyapatite phase, referring to the (002), (211), (310), (312), (213) and (004) planes, respectively [26,47]. These peaks confirm the hexagonal crystal structure of HAp and the absence of secondary phases in the sample. The image of the hydroxyapatite unit cell is shown in Figure S5 and was simulated using the VESTA 3.5.8 (Visualization for Electronic and Structural Analysis) software, using data obtained post-refinement, and indicates that the crystal structure of HAp is hexagonal with space group P/63m. For the modified materials, HApN1, HApN2, and HApN3, shown in Figure 1a, peaks corresponding to hydroxyapatite were observed, confirming that the crystalline structure was maintained even after incorporation of silylating agents. This result shows that the modification occurred without compromising the crystallographic identity of the material in accordance with previous studies [3,48]. However, a slight displacement and broadening of the diffraction peaks may suggest a reduction in the crystallinity or crystallite size of HAp, a common effect observed after surface modification processes, as reported in a previous study [49]. The X-ray diffractogram for cashew gum aerogels and their nanocomposites with pure and modified hydroxyapatite is shown in Figure 1b and indicates that, in general, all materials have an amorphous character, a typical characteristic of some polymers. Nanocomposite aerogels containing pure and modified hydroxyapatite showed intense peaks in the 2θ ≈ 25° and 35° region, which are associated with the characteristic peaks of hydroxyapatite, indicating the incorporation of the inorganic material into the polymeric matrix.

### 3.2. Fourier Transform Infrared Spectroscopy (FTIR)

**Figure 2:**
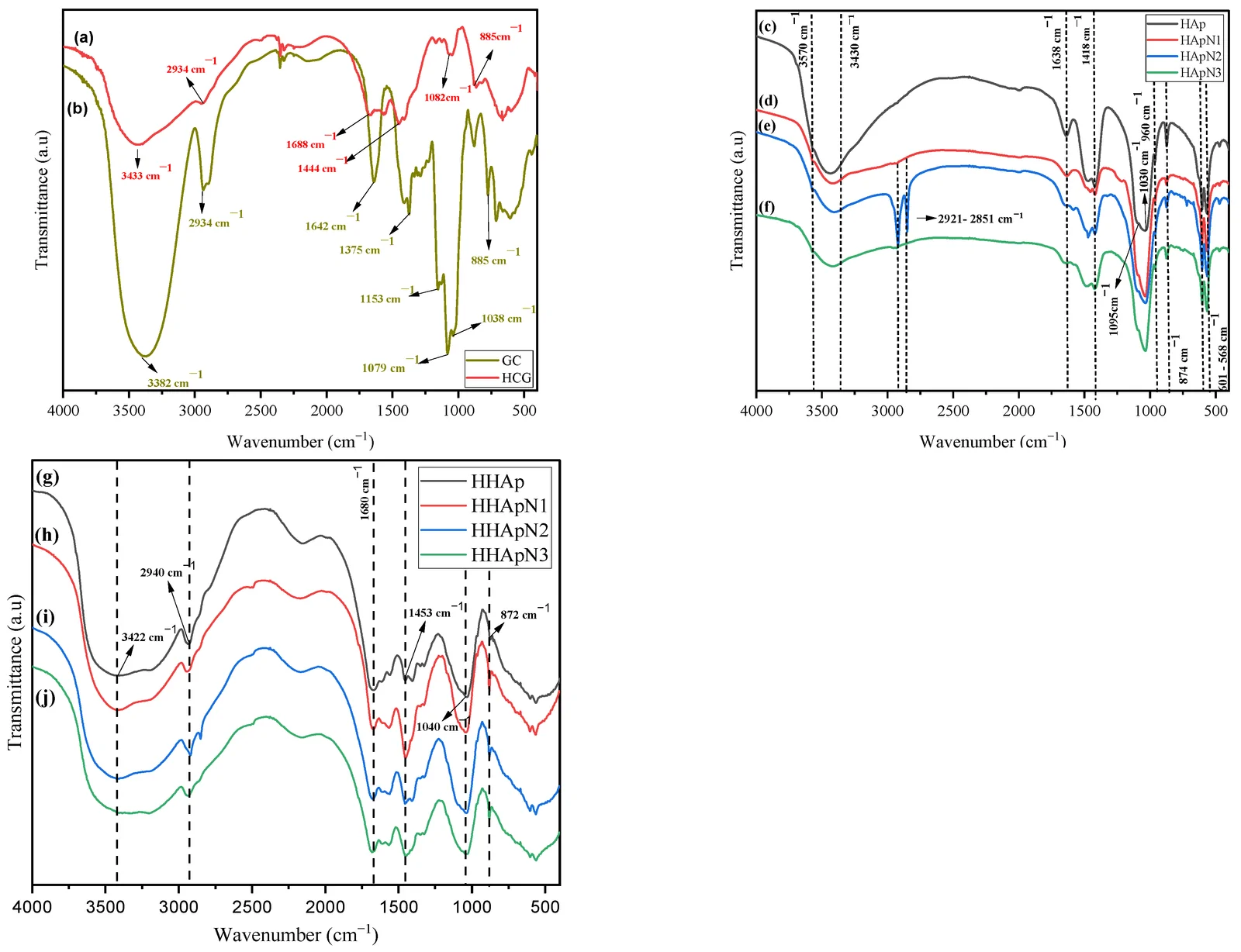
FTIR spectrum for (**a**) cashew gum (CG), (**b**) cashew gum aerogel (HCG), (**c**) HAp, (**d**) HApN1, (**e**) HApN2, (**f**) HApN3, (**g**) HHAp, (**h**) HHApN1, (**i**) HHApN2 and (**j**) HHApN3.

The FTIR spectra of cashew gum and cashew gum aerogel are shown in Figure 2. FTIR spectra confirm the presence of functional groups characteristic of cashew gum, pure and modified hydroxyapatite, and indicates interactions between the polymeric matrix and the inorganic phase.

For cashew gum (CG), the main bands at 3382, 2934, 2890, 1642, 1375, 1153, 1079, 1038, and 885 cm^−1^ were attributed, respectively, to O–H group stretching, asymmetric and symmetric C–H bond stretching, O–H and C–H bond deformation, C–O–C stretching vibrations, O–H deformation, and out-of-plane C–H bending vibration [18,20]. In the HCG aerogel, the band at 1668 cm^−1^ was attributed to C=O stretching resulting from polyacrylamide hydrolysis, while the other bands remained characteristic of the polymer matrix [18,20]. HAp exhibited characteristic bands at 3570 cm^−1^ (O–H bond stretching), 3430 cm^−1^ (adsorbed water), 1638 cm^−1^ (O–H deformation), 1095, 1030, and 960 cm^−1^ (symmetric ${PO}_{4}^{3-}$ stretching), 874 cm^−1^ (P–OH stretching), and 601 and 568 cm^−1^ (O–P–O bond deformation) [3,7,48]. Following aminosilane modification, bands were observed in the 2950–2920 cm^−1^ region, attributed to the CH_2_ stretching of silane groups, along with a shift in the band from 1030 to 1036 cm^−1^, associated with the presence of Si–O–Si siloxane groups [50], which corroborates the functionalization of the HAp. For HHAp, the bands at 3422 and 2940 cm^−1^ were assigned to the O–H and C–H stretching vibrations of the polymer matrix, respectively [29]. The band at 1680 cm^−1^ is associated with N–H or C=O contributions resulting from alkaline hydrolysis [51], while the band at 1453 cm^−1^ corresponds to the asymmetric C=O vibrations of the gum. The band at 1040 cm^−1^ reflects a simultaneous contribution from the C–O–C groups of the gum and the PO_4_^3−^ groups of the HAp, indicating band overlap. The band at 872 cm^−1^ refers to the O–H stretching or the C–H bond stretching of the polysaccharide [52,53]. In materials containing modified HAp, the decrease in O–H band intensity, combined with the presence of silane C–H bands at approximately 2940 cm^−1^, further corroborated the surface modification of the inorganic phase [29]. The shift and broadening of the band at 1453 cm^−1^, observed with increasing aminosilane content, may also be related to the overlap of bands from the polymer matrix and the aminosilane C–H groups [54]. Overall, the FTIR results confirm the presence of the different phases and provide evidence of the HAp modification and its interaction with the polymer matrix.

### 3.3. CHN Elemental Analysis

In CHN elemental analysis, Table S1, for the modified hydroxyapatite precursor materials (HApN1-3), a progressive increase in carbon and nitrogen content was observed as the amount of silylating agents is incorporated into the inorganic matrix [55], with values corresponding to 2.63, 3.35 and 3.68 mmol g^−1^ for C and 0.82, 1.30, and 1.43 mmol g^−1^ for N for HApN1, HApN2 and HApN3, respectively, indicating a good amount of anchored molecules in accordance with studies in the literature [3,55]. The CHN results demonstrate the efficient incorporation of aminosilanes into hydroxyapatite, observed by the progressive increase in carbon and, especially, nitrogen content in the HApN1-3. This behavior confirms that larger quantities of aminosilanes were anchored to the inorganic matrix as the degree of modification increased. Among the aerogels, cashew gum aerogel (HCG) showed the highest carbon and hydrogen contents (% and mmol g^−1^), as expected given its completely organic composition. Furthermore, a higher nitrogen content was also observed, associated with the presence of nitrogenous groups originating from reagents used during the synthesis of the material, which participate in polymerization and the formation of the crosslinked network. With the incorporation of hydroxyapatite, a reduction in the C, H, and N values in HHAp can be observed, a fact that can be attributed to the presence of a higher proportion of the inorganic component in the polymeric matrix of the aerogel. Regarding HHApN1-3 aerogels, a gradual increase in nitrogen content can be observed, a result that is consistent with the increase in aminosilane groups incorporated into the hydroxyapatite after modification, as well as nitrogenous groups from the reagents used in the synthesis. The carbon content for these materials also shows variations consistent with the modification of the inorganic matrix. Therefore, these results suggest that hydroxyapatite was modified, and that the aerogels, in turn, respond to different inorganic compositions, reflecting variations in the distribution of carbon, hydrogen, and nitrogen. Figure S6 shows the proposed structure of hydroxyapatite modified with different aminosilanes.

### 3.4. Thermogravimetric Analysis

**Figure 3:**
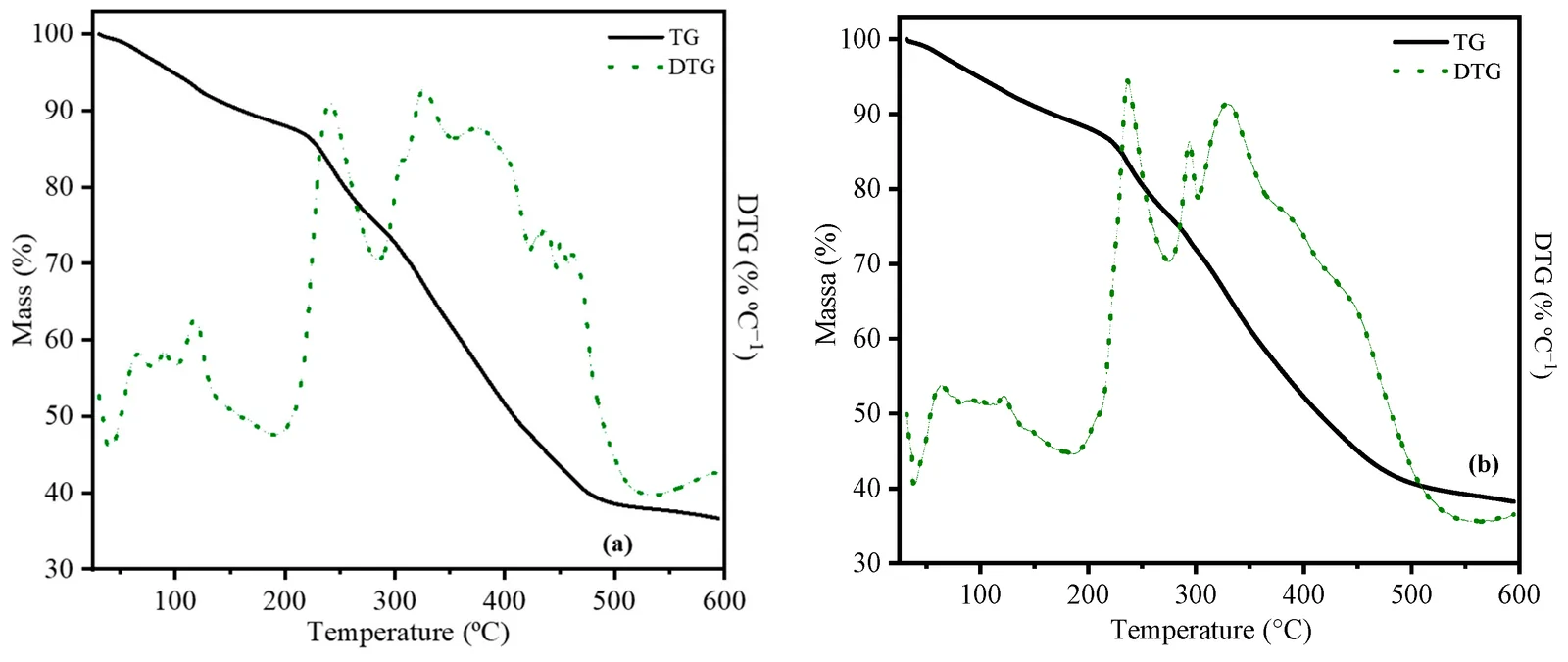

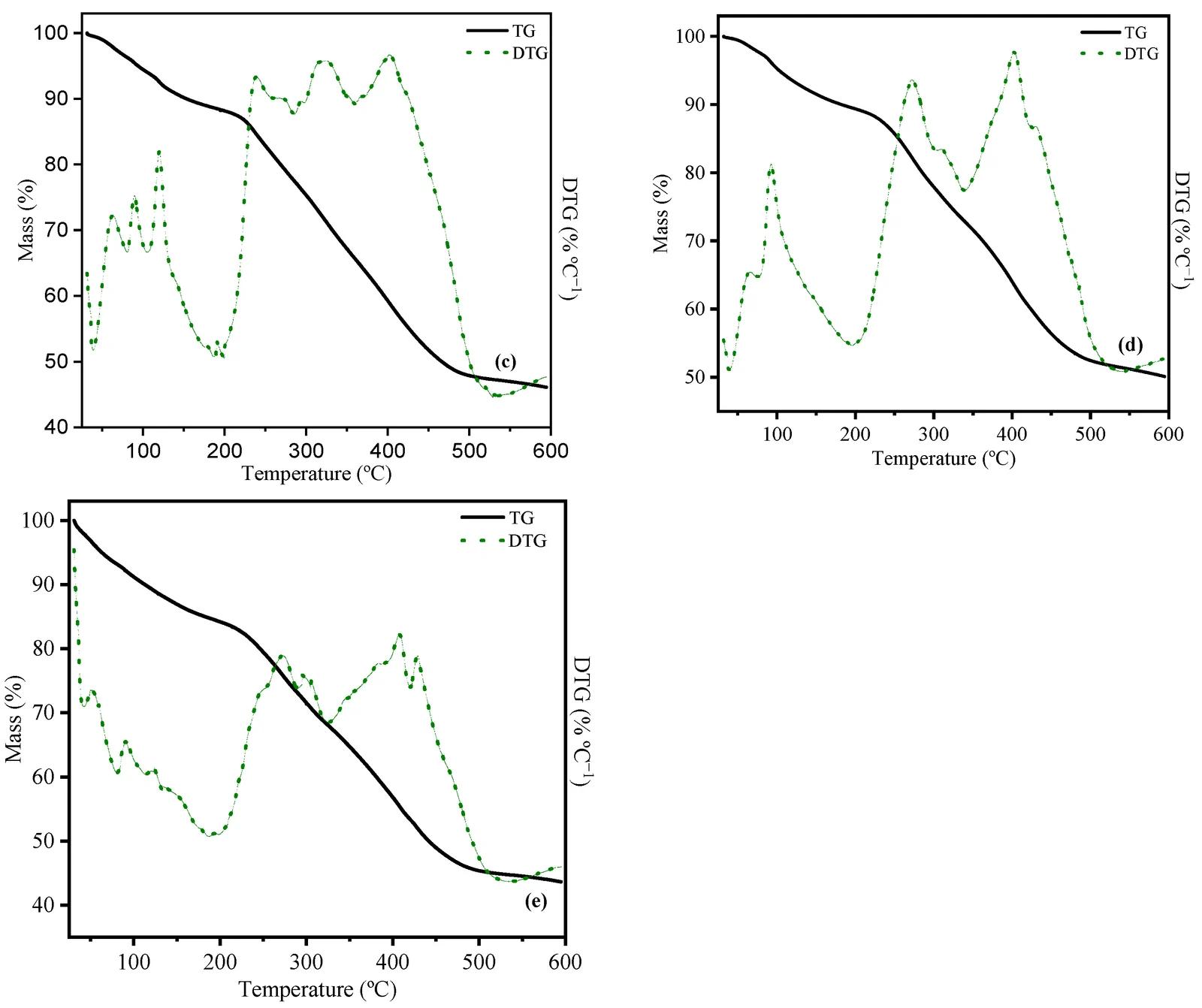
TG and DTG curves for aerogels: (**a**) HCG, (**b**) HHAp, (**c**) HHApN1, (**d**) HHApN2, and (**e**) HHApN3.

The TG/DTG analyses revealed differences in the thermal behavior of the precursor materials (Figure S7) and the aerogels (Figure 3).

Cashew gum (CG) exhibited an initial water loss at approximately 62.7 °C (3%), followed by the main polysaccharide decomposition stage between 256 and 308.5° (47.6%) [18]. Pure HAp exhibited a loss of adsorbed water around 62.1 °C (1.3%) and an event at approximately 457 °C (10.4%), associated with the condensation of structural –OH groups and carbonates generated during the synthesis process [3,26]. The modification of HAp with aminosilanes altered its thermal profile, with additional events associated with the decomposition of the introduced organic chains. For HApN1, HApN2, and HApN3 the additional events attributed to the decomposition of the aminosilane organic chains corroborate the surface modification of the hydroxyapatite [3]. For better visualization, the temperature and mass loss (%) data for each thermal event and material are presented in Table S2. In the thermogravimetric curves of HCG, the events between 66 and 241 °C, with a mass loss of 16.7%, are associated with the elimination of water and volatile compounds [18,29]. The main mass loss (37.6%), between 325 and 436 °C, corresponds to the decomposition of polysaccharide chains and the breakdown of the aerogel’s three-dimensional network, whereas the events between 450 and 460 °C are related to the decomposition of remaining residues [29]. The incorporation of HAp modified this behavior: HHAp exhibited events associated with water removal and the progressive decomposition of the organic fraction, with a total mass loss of 33.8% and the formation of a thermally stable inorganic residue [38]. For HHApN1, the mass losses between 63–120 °C (7.5%) and 192–270 °C (12.9%) were attributed to the elimination of physically adsorbed water and the onset of gum decomposition, whereas the events between 296 and 402 °C (20.7%) corresponded to the decomposition of the polysaccharide matrix and the organic groups of the modified Hap [18,56]. HHApN2 exhibited events associated with the decomposition of aminosilanes and the gum between 270 and 402 °C, as well as an oxidation step for carbonaceous residues above 420 °C [18,56]. For HHApN3, the events between 123 and 305 °C were associated with the decomposition of organic groups derived from the gum and aminosilanes, whereas the mass loss at 408 °C was attributed to the oxidation of carbonaceous residues [56,57]. The final residues of approximately 55%, 50%, and 56.5% for HHApN1, HHApN2, and HHApN3, respectively, confirm the contribution of the inorganic phase and highlight differences in the composition of the nanocomposites. Manzoor et al. [58], for example, produced hydroxyapatite-reinforced pectin hydrogel films (PEC/PVA/APTES/HAp), and the incorporation of HAp increased thermal stability and the final residue due to the contribution of the thermally stable mineral phase. The authors also identified mass loss stages associated with water elimination and the progressive decomposition of the polymer matrix, while APTES contributed to degradation events at higher temperatures. Table S3 presents data on temperatures and mass loss (%) for each event across all aerogels.

### 3.5. Scanning Electron Microscopy (SEM)

**Figure 4:**
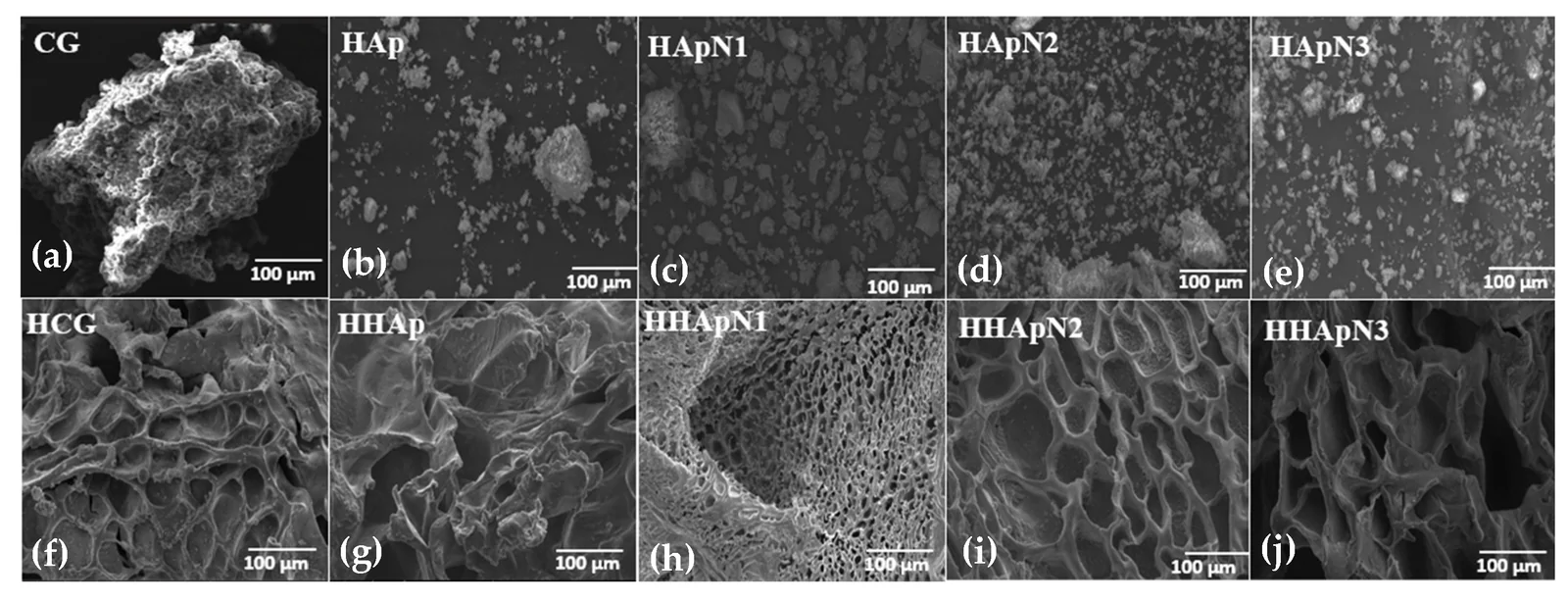
SEM micrographs of the precursor materials (**a**) cashew gum (CG), (**b**) pure hydroxyapatite (HAp), (**c**) HApN1, (**d**) HApN2 and (**e**) HApN3; and the aerogel material (**f**) HCG, (**g**) HHAp, (**h**) HHApN1, (**i**) HHApN2 and (**j**) HHApN3.

Micrographs, Figure 4a, show that cashew gum has a compact and rough structure, while hydroxyapatite, Figure 4b, presents irregular and loosely packed particles. Modification of hydroxyapatite with aminosilanes, Figure 4c–e promoted changes in particle agglomeration and distribution, resulting in a surface coating. Aerogels, Figure 4f–j presented surfaces with cavities, roughness, and discrete pores of irregular sizes and shapes.

### 3.6. Swelling Behavior of Hydrogels

**Figure 5:**
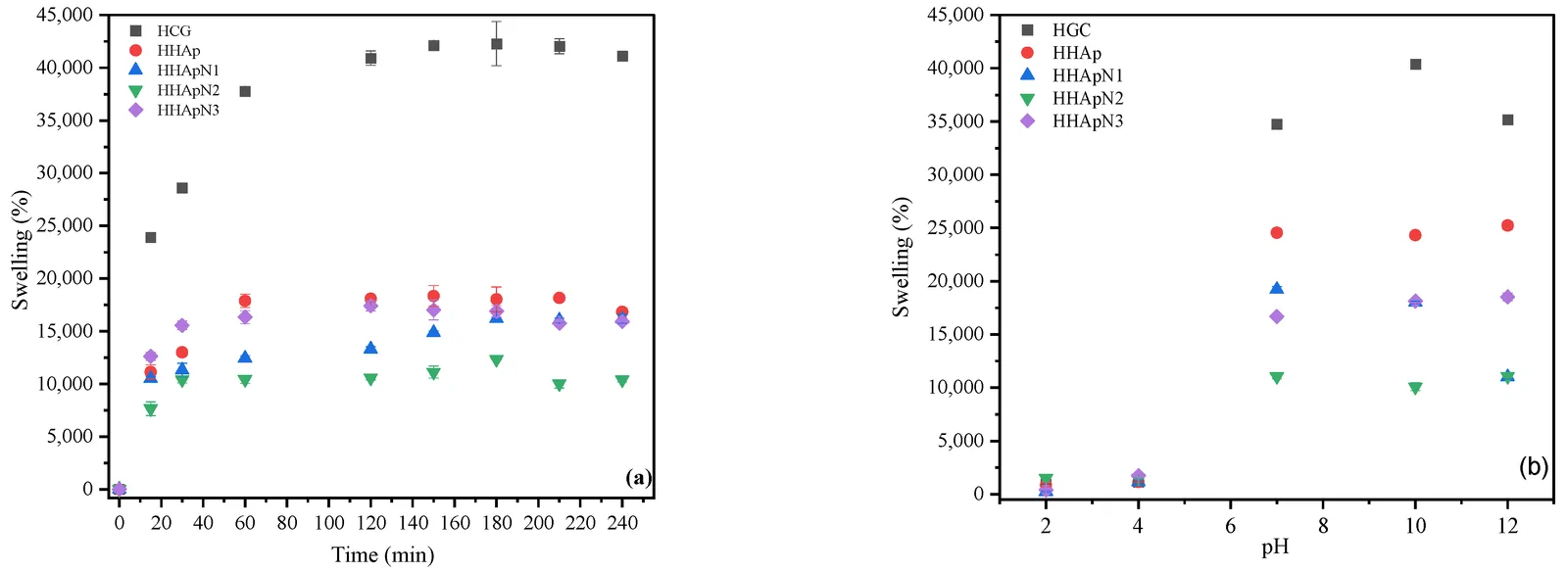
Evaluation of the swelling profile in water as: (**a**) time parameter (from zero to 240 min, at neutral pH) and (**b**) pH parameter (at equilibrium, from pH 2 to 12).

Figure 5a illustrates the swelling in water (neutral pH) for hydrogels over a time interval of 240 min and in Figure 5b, the swelling profile of the hydrogels under study, evaluated over the pH range of 2 to 12.

The hydrogels exhibited swelling rates ranging from 10,000 to 40,000%. Equilibrium time ranged from 60 to 180 min. HCG showed a swelling rate of approximately 40,000%, reaching equilibrium in approximately 150 min. This result may be related to the high hydrophilic capacity of the polymeric matrix [18]. The incorporation of pure hydroxyapatite into the hydrogel matrix influenced HHAp behavior, with a swelling rate of around 18,000% and an equilibrium time of 60 min. This behavior may be related to increased crosslinking and interactions between hydroxyapatite and the polymeric network, favoring a more compact structure and decreasing swelling capacity [29]. For hydrogels containing modified HAp, it was observed that HHApN1 reached equilibrium in 180 min, while HHApN2 and HHApN3, reached it in 60 min, with swelling rates around 16,000%, 10,000%, and 17,000%, respectively. This result may be associated with the modification process, in which the aminosilane groups introduced on the HAp surface favor additional bonding to the polymer matrix, thereby increasing crosslinking. Thus, the presence of modified HAp influences the swelling profile of the materials, restricting the excessive expansion of the polymer network in contact with water and resulting in a more rigid hydrogel, which can be advantageous for applications requiring greater structural integrity and durability in aqueous media. In Figure 5b, the swelling profile of the hydrogels under study, shows that the materials swelled better at neutral and basic pH. It is observed that all hydrogels showed low swelling at more acidic pHs (2 and 4). Under acidic conditions (pH 2–4), all hydrogels exhibited a lower degree of swelling due to the protonation of carboxylate (${\text{–}COO}^{-}$ → –COOH) and amino groups; this promotes hydrogen bonding, reduces electrostatic repulsion between polymer chains, and restricts network expansion [18,59]. As the pH increased, the ionization of functional groups and the resulting increase in electrostatic repulsion favored network expansion and, consequently, swelling [18,60]. The materials showed similar swelling behavior between pH 7 and 12, except for HCG, which exhibited greater swelling at pH 10. Among the nanocomposites containing modified HAp, HHApN2 showed the least swelling under neutral and basic conditions, whereas HHApN3 exhibited the highest swelling capacity under basic conditions. These differences indicate that HAp functionalization influences inter-phase interactions and the pH-responsive swelling behavior of the polymer network. The observed behavior is consistent with studies on cashew gum hydrogels containing an inorganic phase, such as laponite, in which swelling occurs more favorably at more basic pH levels and the addition of the inorganic component reduces swelling due to increased cross-linking density [61].

### 3.7. Study of the Adsorption of Methylene Blue Dye

#### 3.7.1. Contact Time

**Figure 6:**
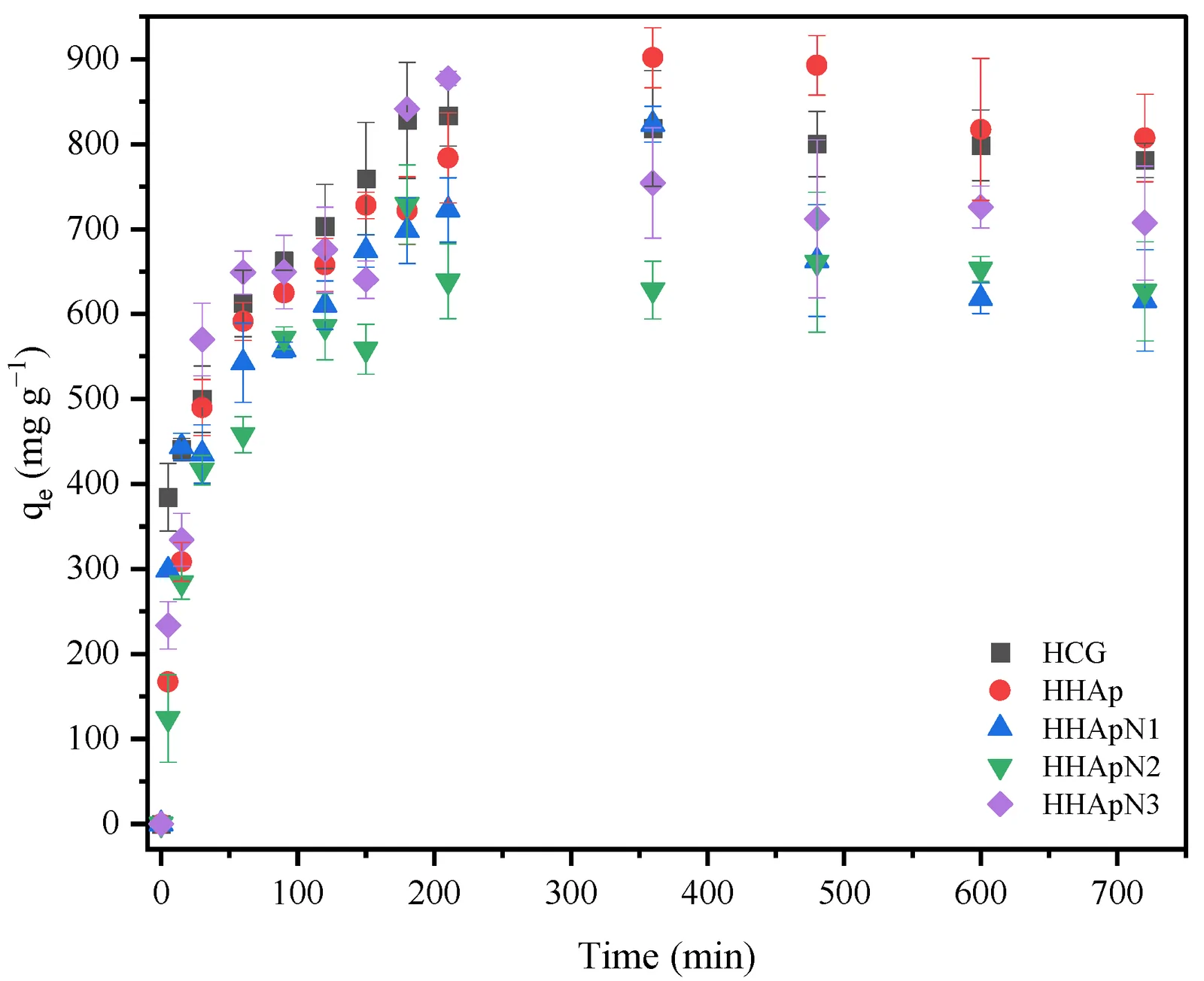
Adsorption kinetics of methylene blue for the different aerogels as a function of contact time. Experimental conditions: adsorbent mass—0.01 g, 30.0 mL of dye solution at a concentration of 500 mg L^−1^, at 25 ± 0.5 °C.

Figure 6 shows the adsorption kinetics graph of the amount adsorbed (q_e_) of methylene blue as a function of contact time (5 to 720 min). The isotherms show that all systems presented an adsorption kinetic profile with a faster phase, which is related to the greater availability of surface active sites and an expansion of the polymer network due to swelling, consequently, the occupation of these by dye molecules, and a slower phase, in which there is a decrease in adsorption due electrostatic repulsion caused by the adsorption of more dye molecules [3]. The systems reached equilibrium in approximately 180 min, after which the variation in the amount adsorbed became constant. Cashew gum aerogel (HCG) exhibited an adsorption capacity of approximately 850 mg g^−1^ for methylene blue. This may be related to the presence of hydrophilic groups in the aerogel’s polymeric matrix, which provides greater swelling. The results in an increase in the surface area of the adsorbent material, providing greater availability of active sites and, consequently, a more effective removal of the pollutant in question [62]. The HHAp aerogel showed an adsorption rate of around 630 mg g^−1^, while the aerogels containing modified hydroxyapatite, HHApN1, HHApN2, and HHApN3, adsorbed approximately 640, 750, and 620 mg g^−1^, respectively. The higher adsorption capacity of HHApN2 suggests that an intermediate degree of modification may favor the balance between amino group availability and accessibility to surface sites, whereas more extensive modification may alter the organization of the organic phase and limit accessibility to adsorption sites [49,62]. HAp incorporation may also restrict the expansion of the polymer network, which could have contributed to the differences observed among the materials [29]. The kinetic data were fitted to the pseudo-first-order (PFO), pseudo-second-order (PSO), and Elovich models (Figure S8). With the exception of HCG, for which the Elovich model yielded a higher R^2^, indicating that adsorption can vary with time and that the adsorbent surface is heterogeneous in which important chemical interactions occur between adsorbent and adsorbate [11], all other systems showed higher R^2^ values for the PFO model (Table S4), which assumes that the adsorption process occurs through physisorption, whose chemical interactions are relatively weak between the adsorbed molecules and the surface of the solids [10]. Furthermore, it is considered a reversible process, and adsorption can occur in a monolayer or multilayer [63]. The observed differences may be related to the structural characteristics of the aerogels. The HGC exhibited greater swelling, which facilitates the expansion of the polymer network and the exposure of the gum’s functional groups, potentially resulting in a greater availability of interaction sites. Conversely, the incorporation of hydroxyapatite, pure or modified, limits network expansion and alters the accessibility of functional groups, creating a more rigid structure where physical interactions between methylene blue and the available sites may predominate. Huang et al. [64] developed an agar/alginate aerogel for methylene blue adsorption and observed that the incorporation of cross-linked alginate reduced swelling due to the formation of a more compact structure and strong hydrogen-bonding interactions. The reduction in network expansion hindered water diffusion and, consequently, limited methylene blue’s access to the adsorption sites.

It was also observed that the theoretical and experimental qe values for the PFO model are close, indicating that this model adequately describes the data. Furthermore, the high k_1_ values for HHApN2 and HHApN3, when compared to HHApN1, may have been influenced by the number of aminosilane groups present in the structure of these materials.

#### 3.7.2. Effect of pH

**Figure 7:**
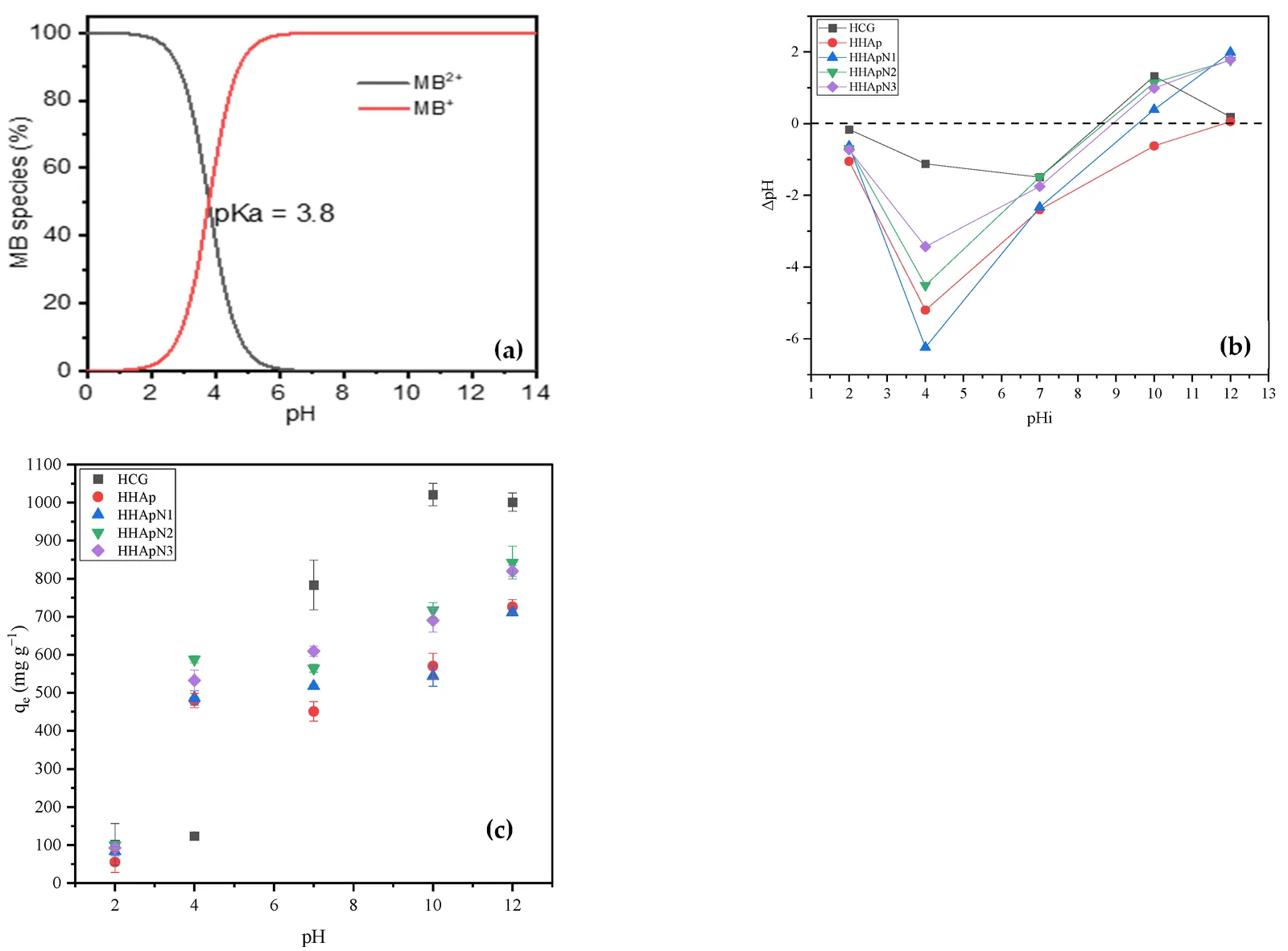
(**a**) Speciation graph of methylene blue dye as a function of pH, (**b**) pHpcz for aerogels, (**c**) effect of pH on the adsorption of methylene blue by aerogels. Experimental conditions: contact time—180 min, adsorbent mass—0.01 g, 30.0 mL of dye solution at a concentration of 500 mg L^−1^, at 25 ± 0.5 °C.

pH investigation is of paramount importance in adsorption studies, as it impacts the surface charge of active sites and the distribution of the dye in aqueous solution, influencing adsorption efficiency [65]. According to the speciation plot for methylene blue, Figure 7a, it can be present in solution as the MB^2+^ or MB^+^ species. The MB^2+^ species predominates at pH < 6, while at pH > 6, the predominant species in solution is MB^+^. Furthermore, when the pH equals the dye’s pKa, both species are present in the medium. The speciation plot indicates that methylene blue remains predominantly in its cationic form throughout the studied pH range. Thus, the adsorption process will be strongly influenced by the surface charge of the adsorbents, as observed in pHpcz, Figure 7b, where the materials exhibited a negative surface charge at pH > pHpcz. The pHpcz values for HCG, HHAp, HHApN1, HHApN2, and HHApN3 were 8.6, 11.7, 9.5, 8.8, and 8.9, respectively. Analyzing the graph in Figure 7c, it is possible to infer that the adsorption capacity of methylene blue increases with increasing pH for all systems under study, reaching values close to 1000 mg g^−1^ for HCG, 720 mg g^−1^ for HHAp, 710 mg g^−1^ for HHApN1, 840 mg g^−1^ for HHApN2 and 820 mg g^−1^ for HHApN3. Overall, methylene blue adsorption was lower at pH 2 due to the protonation of ${\text{–}COO}^{-}$ and –NH_2_ groups, greater shrinkage of the polymer network, and competition from $H^{+}$ ions for adsorption sites [29,66]. Furthermore, the protonation of amino groups (${\text{–}NH}_{3}^{+}$) in the modified HAp promotes electrostatic repulsion with the cationic dye [67]. As the pH increased, a gradual rise in adsorption was observed, particularly in materials containing modified HAp. At neutral pH, the deprotonation of carboxylic groups (${\text{–}COO}^{-}$) favors electrostatic attraction with methylene blue, while increased network hydration may facilitate dye diffusion [38]. Under basic conditions (pH 10–12), adsorption was more pronounced, reaching maximum values at pH 12. In this medium carboxylic groups are predominantly deprotonated, and the silane amino groups exist predominantly in the neutral form (–NH_2_), favoring interaction with the cationic dye [16]. These results agree with the swelling results at more basic pHs, where water absorption rates are higher in this medium, suggesting greater opening of the aerogel polymer network and facilitating the diffusion of methylene blue into the material’s structure. Furthermore, these results are consistent with those observed by Mohammadzadeh et al. [68] in polysaccharide hydrogels, where an increase in MB adsorption with the increase in pH. This behavior was attributed to electrostatic interactions and increased site availability resulting from the deprotonation of carboxylic groups in a basic medium; this process promotes network expansion and the availability of adsorption sites, thereby leading to greater methylene blue removal.

#### 3.7.3. Effect of Concentration

The equilibrium isotherms of the aerogels for methylene blue adsorption were obtained at optimal pH and contact time for each aerogel, over an initial dye concentration range of 10 to 1500 mg L^−1^. The experimental data were fitted to the Langmuir, Freundlich, Temkin, and Dubinin-Radushkevich (D-R) isothermal models. The maximum adsorption capacities were 1103.98, 873.85, 884.64, 505.87, and 625.76 mg g^−1^ for the aerogels HCG, HHAp, HHApN1, HHApN2, and HHApN3, respectively, and the data were best described by the Langmuir and Dubinin-Raduskevich models, respectively. Equilibrium isotherm models are presented in Figure S9, and the isotherm data are presented in Table S5. The results for HCG, HHAp, HHApN1, and HHApN3 best fit the Langmuir model, with higher R^2^ correlation coefficients than those of the other models. The R^2^ values for HGC, HHAp, HHApN1, and HHApN3 were 0.98083, 0.96814, 0.97017, and 0.95152, respectively. The Langmuir model assumes that adsorption occurs in monolayers with a limited number of adsorption sites in the material, each with equivalent energy and without lateral interactions between molecules, resulting in the formation of a single adsorption layer [29]. For HHApN2, the result was described by the Dubinin-Raduskevich (D-R) model, with R^2^ = 0.97197. This model states that adsorption occurs on heterogeneous surfaces, determining the nature of adsorption based on the average potential energy, and describes adsorption in porous materials, where physical adsorption predominates [29]. It is especially useful, for example, for systems with multiple adsorption sites and varying energies [5]. The χ^2^ values are a measure of the agreement of the isotherm model. The smaller the χ^2^ value, the greater the agreement between the model and the data [5]. Therefore, for HCG, HHAp, HHApN1, and HHApN3, the χ^2^ values are smaller for the Langmuir model than for the Freundlich, Temkin, and D-R models, suggesting a better fit of the data to this model. The χ^2^ values for HGC, HHAp, HHApN1, and HHApN3 were 4666.31, 4854.43, 4658.45, and 3853.42, respectively. The χ^2^ value for HHApN2 is better suited to the D-R model and was 1583.53. Furthermore, these results were compared with other aerogel systems and other materials for methylene blue removal (Table 1, indicating the excellent performance of the materials proposed in this study.

#### 3.7.4. Effect of Temperature

**Figure 8:**
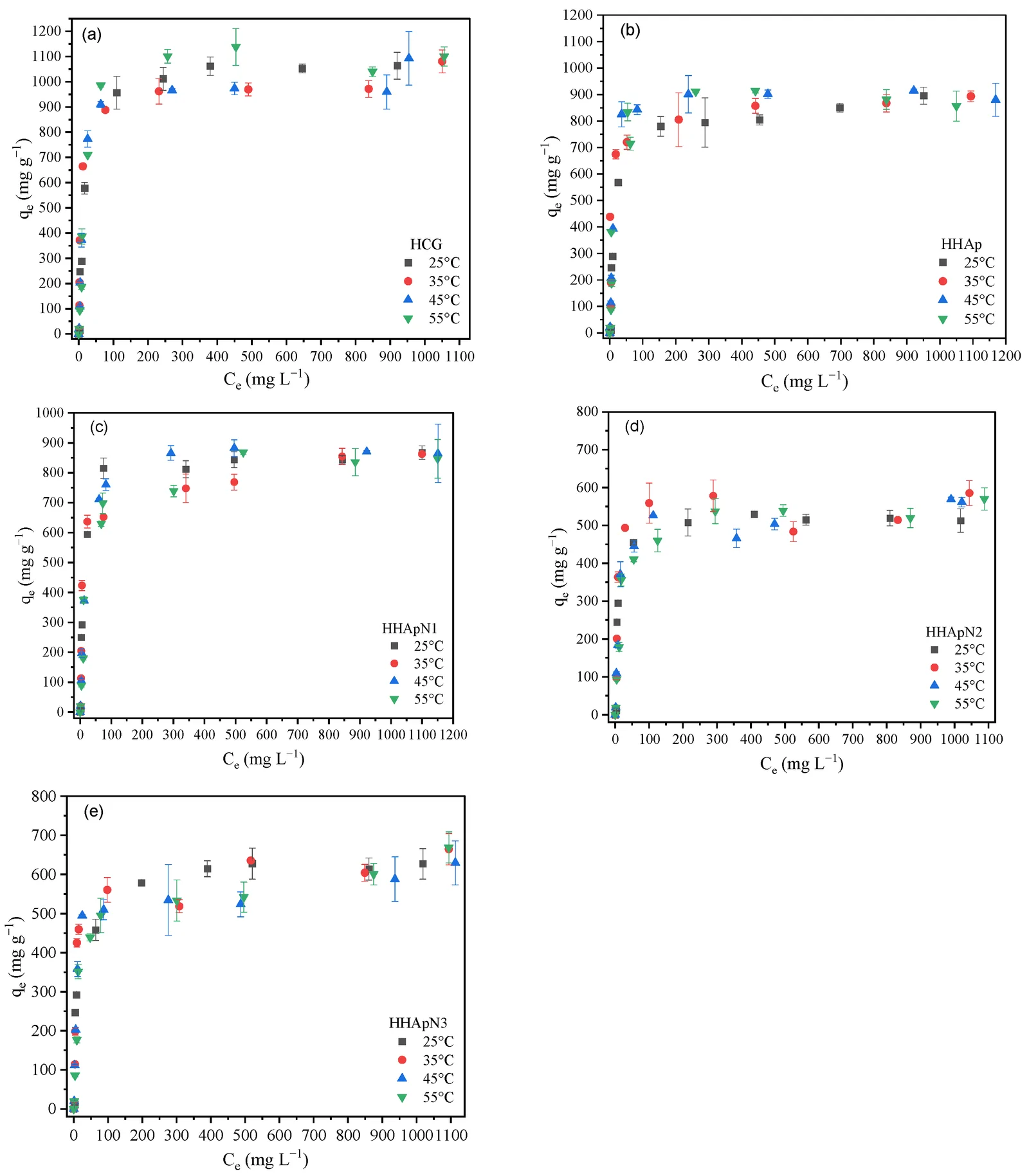
Effect of temperature on the adsorption of methylene blue (MB) onto (**a**) HCG, (**b**) HHAp, (**c**) HHApN1, (**d**) HHApN2, and (**e**) HHApN3 at 25, 35, 45, and 55 °C. Experimental conditions: contact time—180 min, adsorbent mass—0.01 g, 30.0 mL of dye solution.

Figure 8 shows the adsorption isotherms obtained at 25, 35, 45, and 55 °C for the aerogels. The plots exhibited similar profiles for all materials.

Although slight variations in equilibrium adsorption capacity were observed across the different temperatures for the aerogels, there was no significant increase or decrease in adsorption as the temperature rose. Therefore, within the investigated range, temperature did not significantly influence the MB adsorption capacity of the HCG, HHAp, HHApN1, HHApN2, and HHApN3 materials, indicating that MB adsorption by the studied aerogels showed low temperature dependence between 25 and 55 °C. Similar behavior has been reported in different MB adsorption systems. Işıkver [74] studied anionic hydrogels based on acrylamide and 2-hydroxyethyl methacrylate for the adsorption of various cationic dyes, including methylene blue, and evaluated temperatures in the 25 to 45 °C range. The results showed that temperature had no significant effect on the amount of dye adsorbed within the investigated range. Youcef et al. [75] observed that temperature (range 25 at 70 °C) did not significantly affect the adsorption of methylene blue (MB) onto algerian palygorskite. Similarly, Gemici et al. [76] did not report a significant effect of temperature (range of 15 to 45 °C) on MB removal using forestry residues, for example.

#### 3.7.5. Characterization of Hybrid/Methylene Blue Aerogels After Adsorption

**Figure 9:**
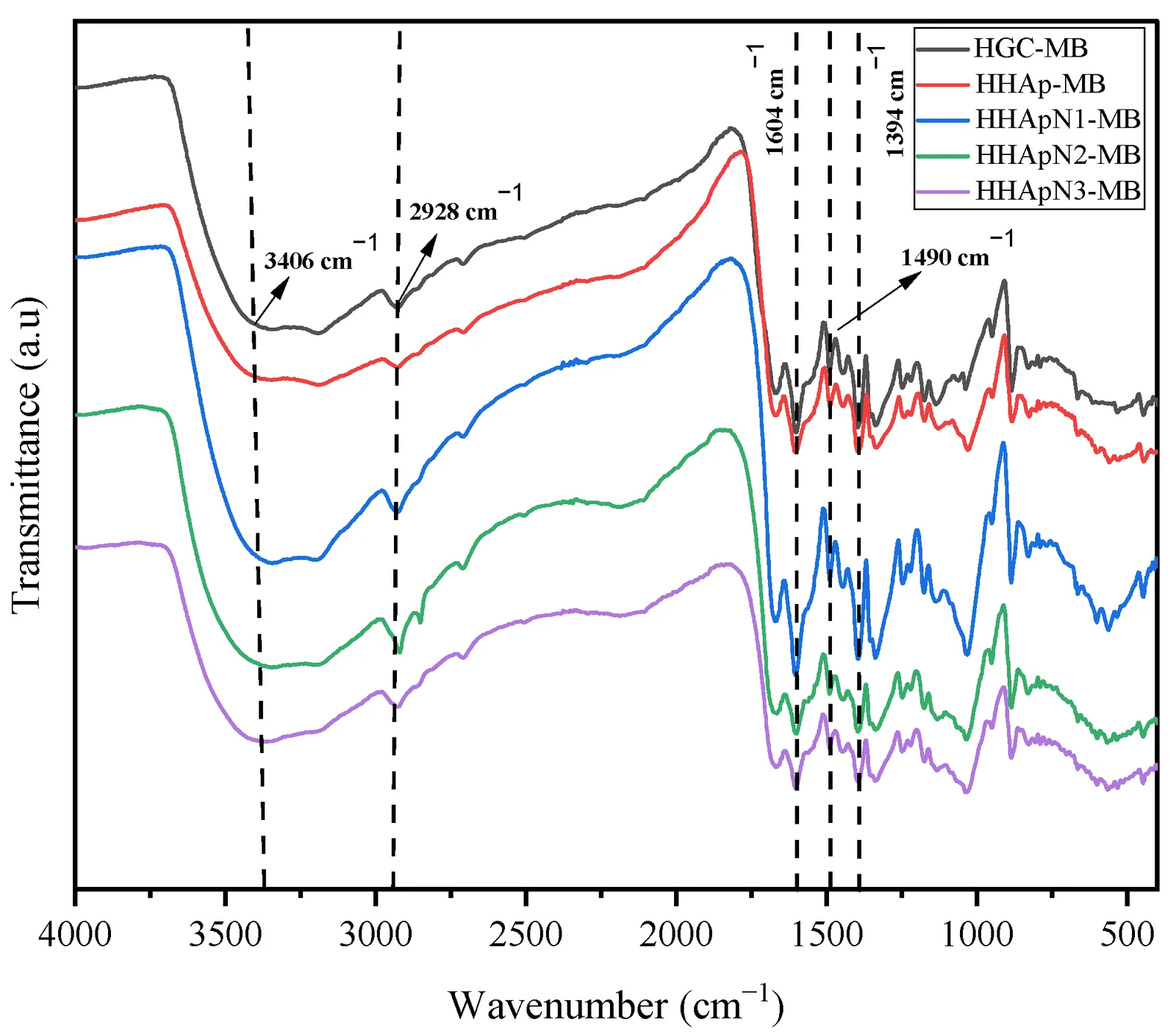
FTIR for the aerogels after adsorption of methylene blue dye.

Figure 9 shows the FTIR spectrum of HCG, HHAp, HHApN1, HHApN2, and HHApN3 aerogels after methylene blue adsorption.

For all aerogels, the bands around 3400 cm^−1^ correspond to O-H stretching bond [77]. The bands around 2928 cm^−1^, 1604 cm^−1^, 1490 cm^−1^ and 1394 cm^−1^, which refers to the asymmetric C-H stretching of the methylene groups, C=C elongation, C=N type bonding in aromatic rings and CH=N stretching or CH_2_ or CH_3_ stretching, respectively [68]. In general, the FTIR spectra of the aerogels after adsorption showed characteristic bands that shifted with changes in intensity. These results may suggest possible interactions between the dye molecules and the functional groups present in the materials. As mentioned earlier, the adsorption of methylene blue must occur through physisorption, where the interactions between the adsorbent and the adsorbate are weak. Electrostatic interactions and hydrogen bonds, for example, may be involved in the adsorption mechanism, as illustrated in Figure S10a [4]. The aerogels were also characterized by SEM. SEM micrographs of the aerogels before adsorption, Figure S10b (images above) show that these materials have a rough surface with disordered cavities and pores. After methylene blue adsorption (images below), some modifications in the morphology of all aerogels can be observed. Greater coverage and modification of the cavities and surface structures were observed. These changes may indicate the deposition of the dye on the surface of the aerogels. These results agree with the adsorption equilibrium isotherms, whose experimental data for HCG, HHAp, HHApN1, and HHApN3 were best fit to the Langmuir model, and HHApN2 to the Dubinin-Raduskevich model. Furthermore, the fact that adsorption for the materials is followed by physisorption reinforces the possibility of the formation of both monolayers and multilayers, which is compatible with the variations in surface coverage observed in SEM images [10,63]. Therefore, physical adsorption must be one of the mechanisms of adsorption for the methylene blue dye.

## 4. Conclusions

In this study, nanocomposite aerogels based on cashew gum and hydroxyapatite (both pristine and aminosilane-modified) were synthesized, and their capacity to remove the dye methylene blue was evaluated. Structural, chemical, thermal, and morphological characterization results confirmed the formation of the materials, the incorporation of hydroxyapatite (HAp) into the cashew gum matrix, and the modification of the HAp with different aminosilane groups. Swelling tests demonstrated that the hydrogels possessed a high-water absorption capacity, reaching equilibrium between 120 and 150 min; the behavior was pH-dependent, with more pronounced swelling rates observed in neutral and basic media. Point of zero charge results indicated that the materials exhibited a predominantly negatively charged surface at pH values above the pHpcz, favoring interaction with cationic species such as methylene blue. This behavior helps explain the higher adsorption capacity observed in basic media, driven by electrostatic interaction with the dye. Kinetic studies showed that adsorption equilibrium was reached at 180 min, with data fitting best to the pseudo-first-order model. Regarding adsorption isotherms, the Langmuir and Dubinin-Radushkevich models provided the best fits. The evaluation of the temperature effect, in turn, showed no temperature dependence of the adsorption under the conditions investigated, indicating that this parameter did not significantly influence the adsorption of methylene blue. Post-adsorption characterization revealed changes in FTIR spectra and the presence of bands associated with the dye, confirming its interaction with the aerogel surface. Overall, the results indicated that the incorporation of hydroxyapatite, and especially its modification with aminosilane groups, influenced the structural, morphological, and adsorption properties of the cashew gum aerogels and their nanocomposites. The developed aerogels proved to be promising materials for effluent decontamination, standing out as sustainable and efficient alternatives for the removal of cationic dyes from aqueous media. However, the mechanical stability of the aerogels in an aqueous environment was not evaluated in this study, nor were their regeneration and reusability. This represents a limitation that should be investigated in future work to assess the materials’ applicability under conditions closer to those of actual water treatment systems.

## Figures and Tables

**Table 1:** Different adsorbent materials were applied in the adsorption of the cationic dye methylene blue.

Adsorbent Material	Experimental Conditions	Amount Adsorbed	Reference
Birch wood pellet biochar	pH 11, 1.020 min	91.0 mg g^−1^	[69]
Hydroxyapatite	pH 7, 20 min	40.0 mg g^−1^	[7]
Magnetic activated carbon	pH 8, 60 min	227.27 mg g^−1^	[13]
Poly(acrylic acid), PAAc and Poly(vinyl alcohol) (PVA) aerogel	pH 6, 400 min	27.0 mg g^−1^	[10]
Hydroxyapatite	pH 12, 4 min	33.33 mg g^−1^	[32]
Ceramic filters	pH 11, 30 min	267.5 mg g^−1^	[70]
Muscovite-kaolinite (Mu-K) clay	pH 8, 60 min	70.93 mg g^−1^	[71]
Calcium phosphate glasses	pH 9, 120 min	66.43 mg g^−1^	[72]
Aerogel agar/k-carrageenan	pH 7, 210 min	242.3 mg g^−1^	[73]
HCG	pH 12, 180 min	1103.98 mg g^−1^	(This work)
HHAp	pH 12, 180 min	873.85 mg g^−1^	(This work)
HHApN1	pH 12, 180 min	884.64 mg g^−1^	(This work)
HHApN2	pH 12, 180 min	505.87 mg g^−1^	(This work)
HHApN3	pH 12, 180 min	625.76 mg g^−1^	(This work)

## Data Availability

The methodology section provides details on the materials used, including the nanocomposite aerogels, and describes how the adsorption tests were conducted. This section contains all the information necessary to reproduce the materials and perform the experiments according to the methods described. In addition, the datasets used and/or analyzed during the study are available upon request from the corresponding author.

## Supplementary Materials

The following supporting information can be downloaded at https://www.mdpi.com/article/10.3390/polym18182306/s1, Figure S1. Structure of aminosilanes (a) 3-aminopropyltrimethoxysilane, (b) 3-propylethylenediaminotrimethoxysilane and (c) 3-propyldiethylenetriaminotrimethoxysilane used in the modification of hydroxyapatite; Figure S2. (a) Exudate extracted from fissures found in cashew trees (*Anacardium occidentale* L.) located at the Federal University of Piauí, and (b) cashew gum obtained after purification; Figure S3. Absorption spectrum of the cationic dye methylene blue; Figure S4. Diffractogram for hydroxyapatite after refinement by the Rietveld method; Figure S5. Unit cell of hydroxyapatite, whose crystal structure is hexagonal (P atoms in lilac, O in red, and Ca in blue); Figure S6. Proposed structures of hydroxyapatite after modification with (a) 3-aminopropyltrimethoxysilane, (b) 3-propylethylenediaminotrimethoxysilane, and (c) 3-propyldiethylenetriaminotrimethoxysilane, in accordance with the experimental C/N ratio; Figure S7. TG and DTG curves for aerogels: (a) Cashew gum, (b) HAp, (c) HApN1, (d) HApN2, and (e) HApN3; Figure S8. Fitting of experimental data to pseudo-first and second order kinetic models and Elovich for nanocomposite hydrogels (a) HGC, (b) HHAp, (c) HHApN1, (d) HHApN2, and (e) HHApN3; Figure S9. Equilibrium isotherms for methylene blue adsorption for nanocomposite hydrogels (a) HCG, (b) HHAp, (c) HHApN1, (d) HHApN2, and (e) HHApN3; Figure S10. (a) Schematic representation of the proposed methylene blue adsorption mechanism by aerogels as a function of the pH of the medium; (b) SEM images of HCG, HHAp, HHApN1, HHApN2, and HHApN3 aerogels before (images above) and after adsorption (images below) of methylene blue; Table S1. CHN elemental analysis of modified hydroxyapatite precursors (HApN1–N3) and cashew gum aerogels (HGC) with pure (HHAp) and modified (HHApN1–N3) hydroxyapatite. The table presents the percentages and molar contents (mmol g^−1^) of C, H, and N, along with the theoretical and experimental C/N ratios; Table S2. Thermogravimetric profile of the precursor materials, cashew gum (CG), HAp, and HApN1-3 with their respective temperatures and mass losses in %; Table S3. Thermogravimetric profile of HCG, HHAp, and HHApN1-3 aerogels with their respective temperatures and mass losses in (%) in each thermal event; Table S4. Parameters of the pseudo-first-order (PFO), second-order (PSO), and Elovich kinetic models for the adsorption of methylene blue by composite materials; Table S5. Adsorption data of methylene blue dye by cashew gum nanocomposite aerogels obtained after fitting to the Langmuir, Freundlich, Temkin, and Dubinin-Raduskevich (D-R) models.

## Abbreviations

The following abbreviations are used in this manuscript:

CG	Cashe gum
HAp	Hydroxyapatite
HApN1	Hydroxyapatite modified with 3-aminopropyltrimethoxysilane
HApN2	Hydroxyapatite modified with 3-propylethylenediaminotrimethoxysilane
HApN3	Hydroxyapatite modified with 3-propyldiethylenetriaminotrimethoxysilane
HCG	Cashew gum aerogel
HHAp	Aerogel nanocomposite cashew gum/hydroxyapatite pure
HHApN1	Aerogel nanocomposite cashew gum/hydroxyapatite modified with 3-aminopropyltrimethoxysilane
HHApN2	Aerogel nanocomposite cashew gum/hydroxyapatite modified with 3-propylethylenediaminotrimethoxysilane
HHApN3	Aerogel nanocomposite cashew gum/hydroxyapatite modified with 3-propyldiethylenetriaminotrimethoxysilane
MB	Methylene blue
