# Supplementary material for: Cashew Gum/Pure and Modified Hydroxyapatite Nanocomposite Aerogels for Water-Pollutant Removal
Source: Polymers (Basel). 2026 Sep 20;18(18):2306. doi: 10.3390/polym18182306 (PMC13611090; doi:10.3390/polym18182306)
Supplement: Supplementary file 1 [file polymers-18-02306-s001.zip › polymers-4424808-supplementary.pdf]

Supplementary material

Cashew Gum/Pure and Modified Hydroxyapatite Nanocomposite Aerogels for Water-Pollutant Removal

Joyce Araujo Borges [1], Ariane Maria da Silva Santos [1], Erica Ianne da Silva Sousa [1], Edvani Curti Muniz [1], Josy Antevelli Osajima [1], Luís Humberto de Oliveira [1], Maria Gardênnia Fonseca [2], Rafael de Carvalho Araújo [2] and Edson C. Silva-Filho [1,*]

[1] LIMAV—Interdisciplinary Laboratory for Advanced Materials, Campus Ministro Petrônio Portella, Federal University of Piauí, Teresina 64049-550, Brazil; joyceab@ufpi.edu.br (J.A.B.); ariane.am42@gmail.com (A.M.d.S.S.); ericaianne123@ufpi.edu.br (E.I.d.S.S.); curtimuniz@gmail.com (E.C.M.); josyosajima@ufpi.edu.br (J.A.O.); luishumbertodeoliveira@gmail.com (L.H.d.O.)

[2] Research and Extension Center-Fuel and Materials Laboratory (NPE–LACOM), Federal University of Paraíba—UFPB, João Pessoa 58059-900, Brazil; mgardennia@gmail.com (M.G.F.); rafaell.ufpb@gmail.com (R.d.C.A.)

* Correspondence: edsonfilho@ufpi.edu.br

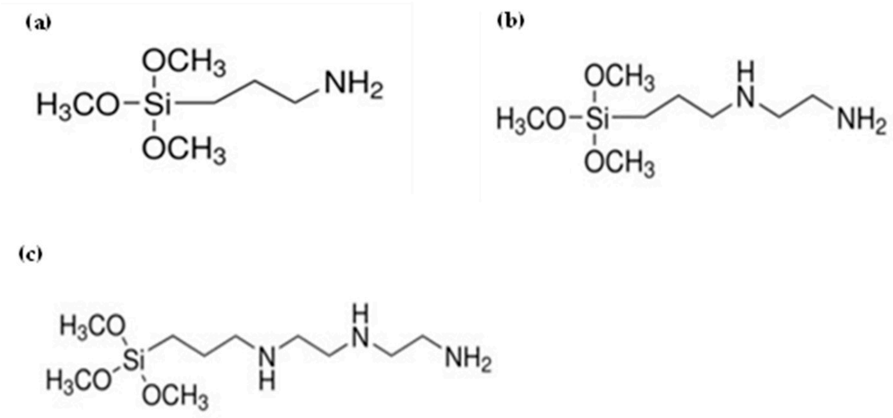

Figure S1. Structure of aminosilanes (a) 3-aminopropyltrimethoxysilane, (b) 3-propylethylenediaminotrimethoxysilane and (c) 3-propyldiethylenetriaminotrimethoxysilane used in the modification of hydroxyapatite

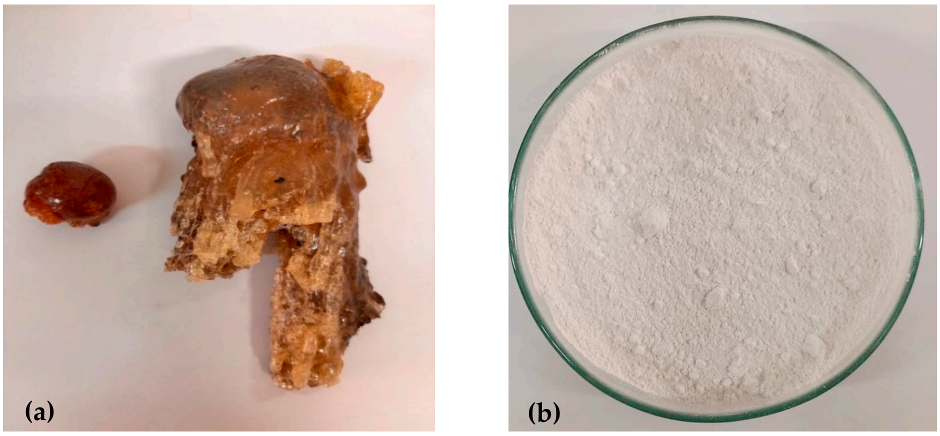

Figure S2. a) Exudate extracted from fissures found in cashew trees (*Anacardium occidentale L.*) located at the Federal University of Piauí, and b) cashew gum obtained after purification.

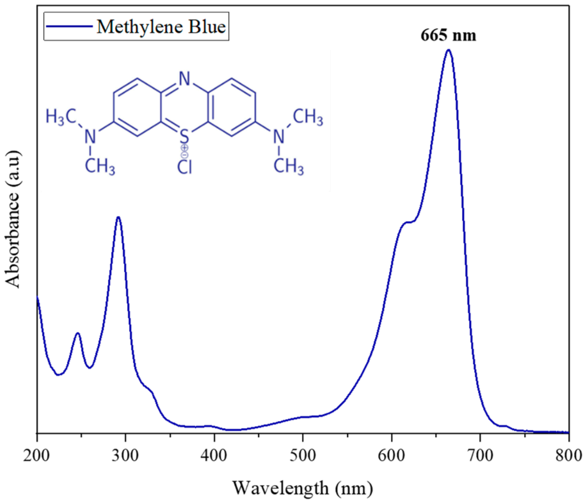

Figure S3. Absorption spectrum of the cationic dye methylene blue.

X-ray diffractometry (XRD)

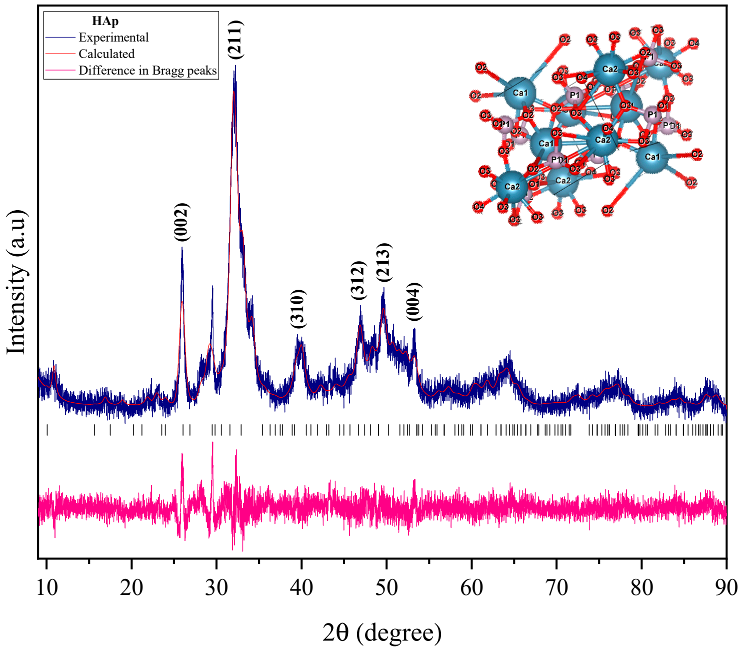

Figure S4. Diffractogram for hydroxyapatite after refinement by the Rietveld method.

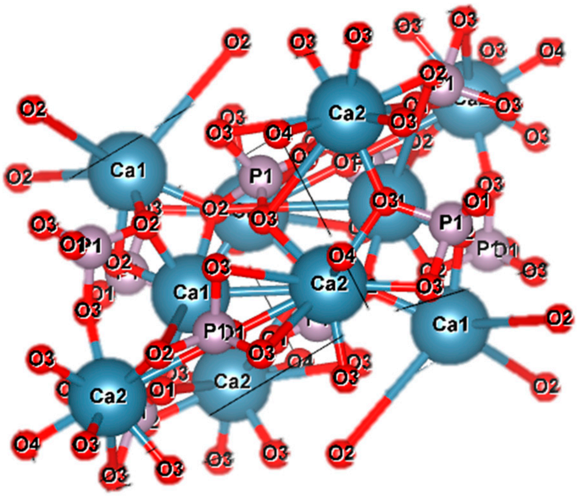

Figure S5. Unit cell of hydroxyapatite, whose crystal structure is hexagonal (P atoms in lilac, O in red, and Ca in blue).

Elemental analysis CHN

Table S1. CHN elemental analysis of modified hydroxyapatite precursors (HApN1–N3) and cashew gum aerogels (HGC) with pure (HHAp) and modified (HHApN1–N3) hydroxyapatite. The table presents the percentages and molar contents (mmol g^{-1}) of C, H, and N, along with the theoretical and experimental C/N ratios.

Sample	Concentration (%)			mmol g^{-1}			C/N_{teo}	C/N_{exp}
	C	H	N	C	H	N		
CG	3.35	-	0.01	2.79	-	0.01	-	-
HApN1	3.16	-	1.14	2.63	-	0.82	3.00	3.21
HApN2	4.03	-	1.81	3.35	-	1.30	2.50	2.59
HApN3	4.42	-	2.01	3.68	-	1.43	2.33	2.57
HGC	39.4	9.3	8.8	32.8	93.8	6.28	-	5.22
HHAp	33.5	7.13	7.10	27.9	71.3	5.07	-	5.50
HHApN1	25.8	4.09	3.60	21.6	40.0	2.57	-	8.40
HHApN2	31.6	7.62	4.54	26.3	76.2	3.24	-	8.12
HHApN3	31.4	7.23	4.90	26.2	72.3	3.50	-	7.50

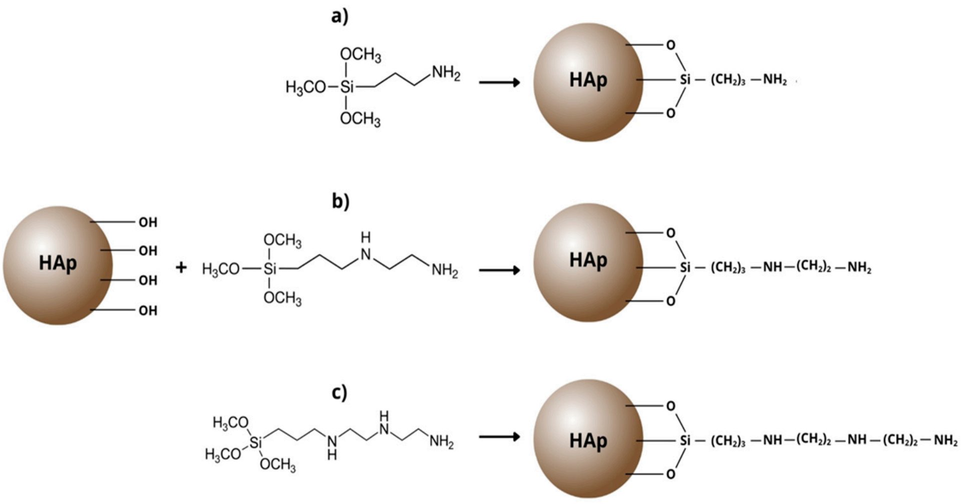

Figure S6. Proposed structures of hydroxyapatite after modification with a) 3-aminopropyltrimethoxysilane, b) 3-propylethylenediaminotrimethoxysilane, and c) 3-propyldiethylenetriaminotrimethoxysilane, in accordance with the experimental C/N ratio.

Thermogravimetric analysis

Table S2. Thermogravimetric profile of the precursor materials, cashew gum (CG), HAp, and HApN1-3 with their respective temperatures and mass losses in %.

Sample	Event/Temperature (ºC)	Mass Loss %
GC	1º event – T ≈ 62.7 °C	3%
	2º event – T ≈ 256 °C	18.3%
	3º event – T ≈ 308.5 °C	29.3%
HAp	1º event – T ≈ 62.1 °C	1.3%
	2º event – T ≈ 256 °C	10.4%
HApN1	1º event – T ≈ 60 °C	2.4%
	2º event – T ≈ 111 °C	4.1%
	3º event – T ≈ 462.3 °C	12.7%
HApN2	1º event – T ≈ 67 °C	0.7%
	2º event – T ≈ 470 °C	15.8%
HApN3	1º event – T ≈ 66 °C	1.2%
	2º event – T ≈ 376.8 °C	9.8%
	3º event – T ≈ 535 °C	4.9%

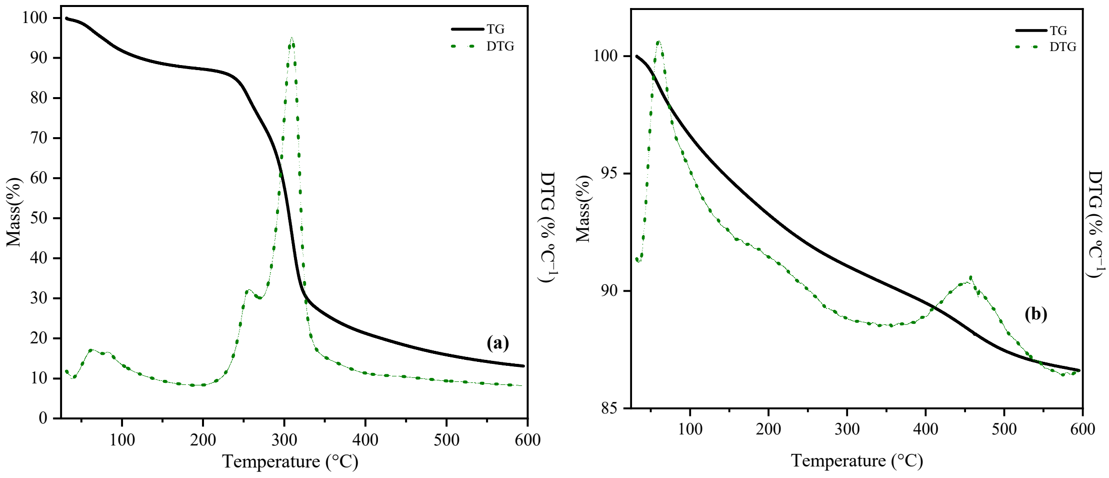

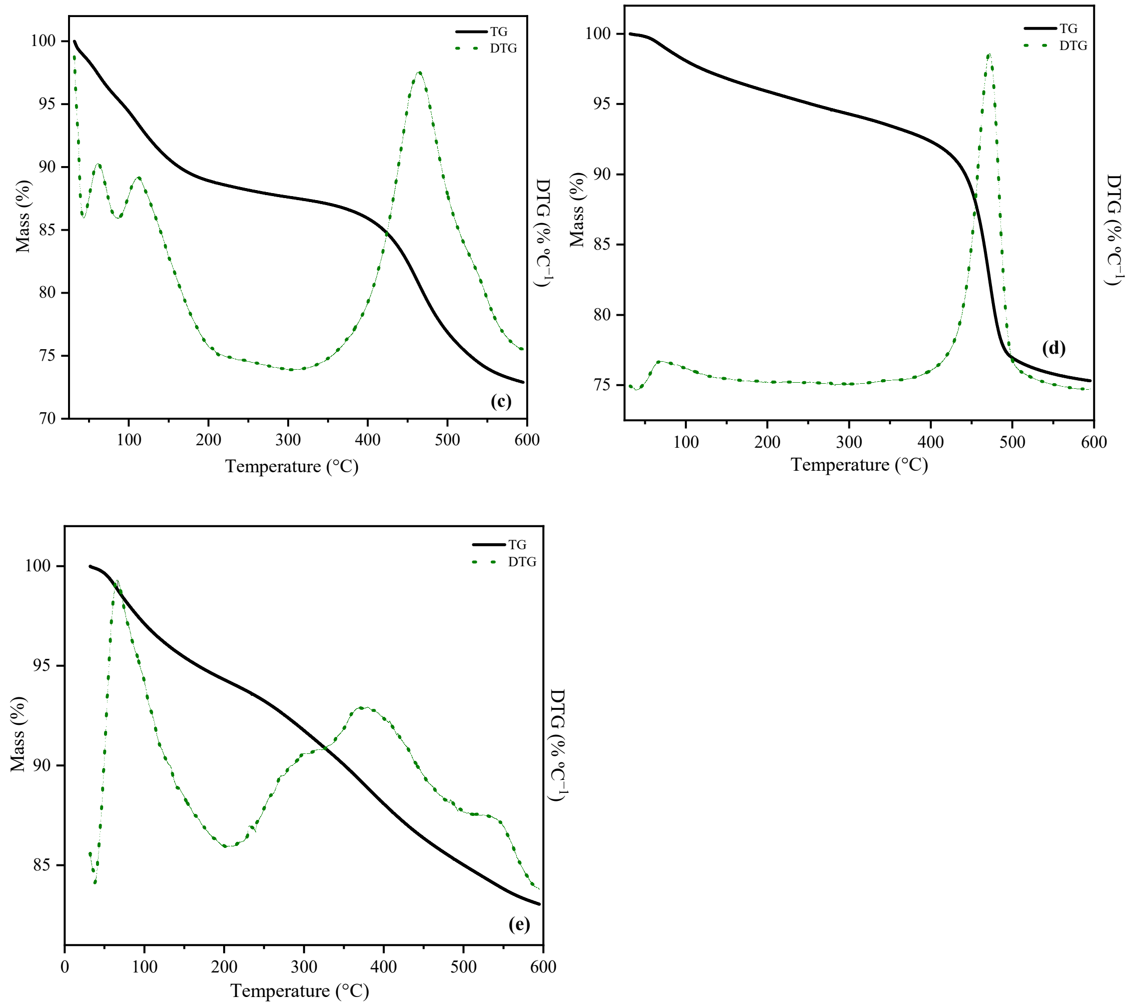

Figure S7. TG and DTG curves for aerogels: (a) Cashew gum, (b) HAp, (c) HApN1, (d) HApN2, and (e) HApN3.

Table S3. Thermogravimetric profile of HCG, HHAp, and HHApN1-3 aerogels with their respective temperatures and mass losses in (%) in each thermal event.

Sample	Event/Temperature (ºC)	Mass loss %
HCG	1º event – T ≈ 66 °C	2.3%
	2º event – T ≈ 90 °C	2%
	3º event – T ≈ 120 °C	2.7%
	4º event – T ≈ 241 °C	9.8%
	5º event – T ≈ 325 °C	15.6%
	6º event – T ≈ 374 °C	10.6%
	7º event – T ≈ 436 °C	11.4%
	8º event – T ≈ 450 °C	2.3%
	9º event – T ≈ 460 °C	1%
HHAp	1º event – T ≈ 63.2 °C	1.9%
	2º event – T ≈ 120 °C	5%

	3º event – T ≈ 236.5 °C	9.4%
	4º event – T ≈ 293 °C	10.5%
	5º event – T ≈ 329 °C	7%
HHApN1	1º event – T ≈ 63.4 °C	2.2%
	2º event – T ≈ 88.5 °C	2.3%
	3º event – T ≈ 119.5 °C	3%
	4º event – T ≈ 192 °C	4.1%
	5º event – T ≈ 238 °C	3.5%
	6º event – T ≈ 270 °C	5.3%
	7º event – T ≈ 296 °C	3.5%
	8º event – T ≈ 322 °C	4.1%
	9º event – T ≈ 402 °C	13.1%
HHApN2	1º event – T ≈ 64 °C	1.5%
	2º event – T ≈ 91.4 °C	7.2%
	3º event – T ≈ 270 °C	8.7%
	4º event – T ≈ 310 °C	5.8%
	5º event – T ≈ 402 °C	13.2%
	6º event – T ≈ 430 °C	4.2%
HHApN3	1º event – T ≈ 50 °C	3.3%
	2º event – T ≈ 90 °C	4.6%
	3º event – T ≈ 123 °C	3%
	4º event – T ≈ 272 °C	13%
	5º event – T ≈ 297 °C	4.1%
	6º event – T ≈ 305 °C	1.2%
	7º event – T ≈ 408 °C	15.4%
	8º event – T ≈ 428 °C	3.4%

Adsorption: contact time

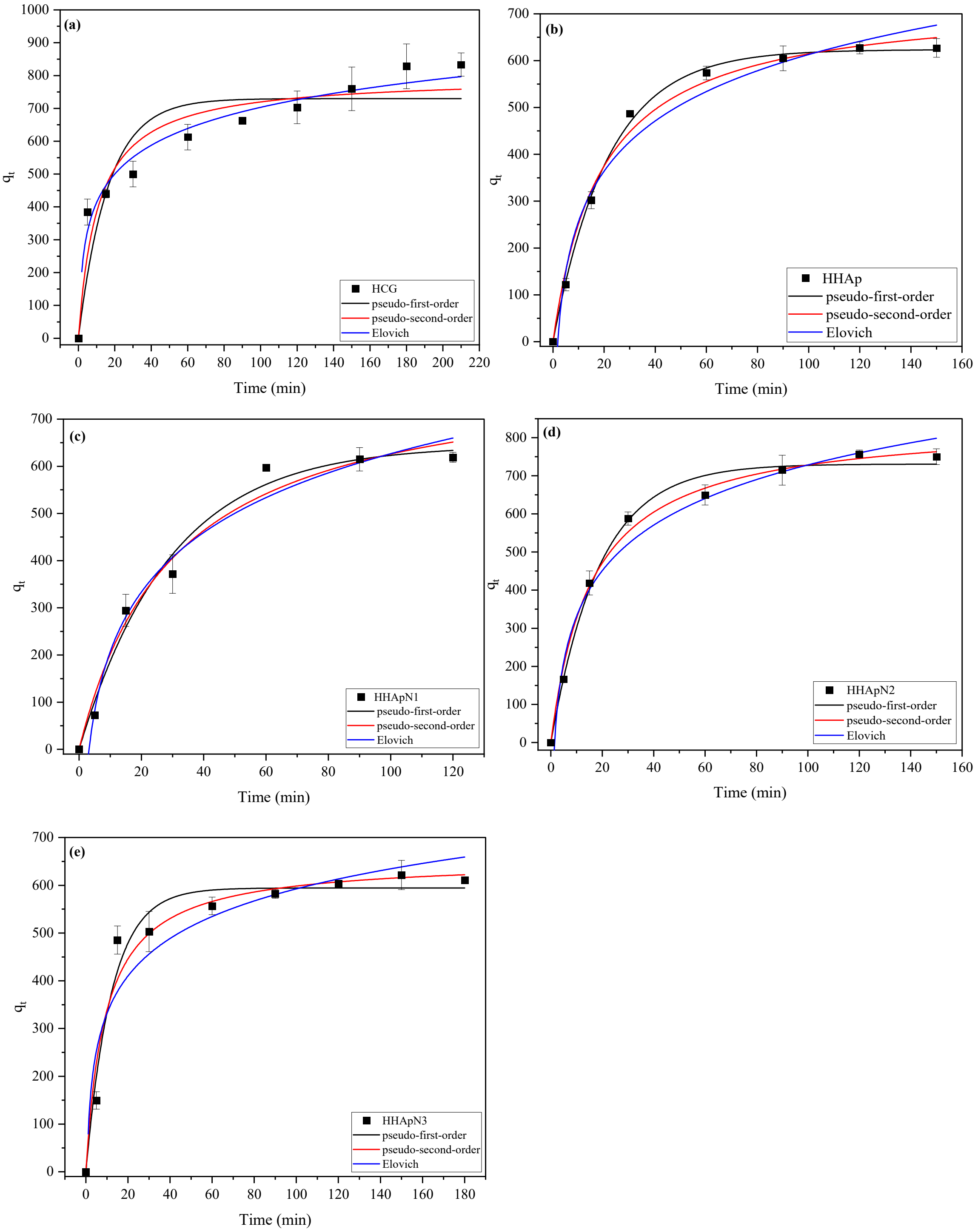

Figure S8. Fitting of experimental data to pseudo-first and second order kinetic models and Elovich for nanocomposite hydrogels (a) HGC, (b) HHAp, (c) HHApN1, (d) HHApN2, and (e) HHApN3.

Table S4. Parameters of the pseudo-first-order (PFO), second-order (PSO), and Elovich kinetic models for the adsorption of methylene blue by composite materials.

Model	Parameter	Sample				
		**HCG**	**HHAp**	**HHApN1**	**HHApN2**	**HHApN3**
**PFO**	k_1(min^{-1})	0.0614	0.0462	0.0342	0.0532	0.0819
	$q_{e(calc)}$ (mg g^{-1})	729.8	623.5	644.2	730.9	594.3
	$q_{e(exp)}$ (mg g^{-1})	729.8	622.8	633.3	730.6	594.4
	R^2	0.853	0.998	0.987	0.994	0,973
**PSO**	k_2(min^{-1})		11.4x10^{-5}	7.18x10^{-5}	7.49x10^{-5}	16.3x10^{-5}
	$q_{e(calc)}$ (mg g^{-1})	797.5	654.1	731.4	762.9	654.4
	$q_{e(exp)}$ (mg g^{-1})	758.3	609.1	648.8	843.7	622.1
	R^2	0.923	0.963	0.991	0.992	0.966
**Elovich**	α (mg g^{-1} min^{-1})	332.4	81.31	56.75	117.8	210.8
	β (10^{-4} g mg^{-1})	0.793	0.646	0.549	0.58	0.882
	R^2	0.978	0.979	0.981	0.983	0.931

Adsorption isotherms

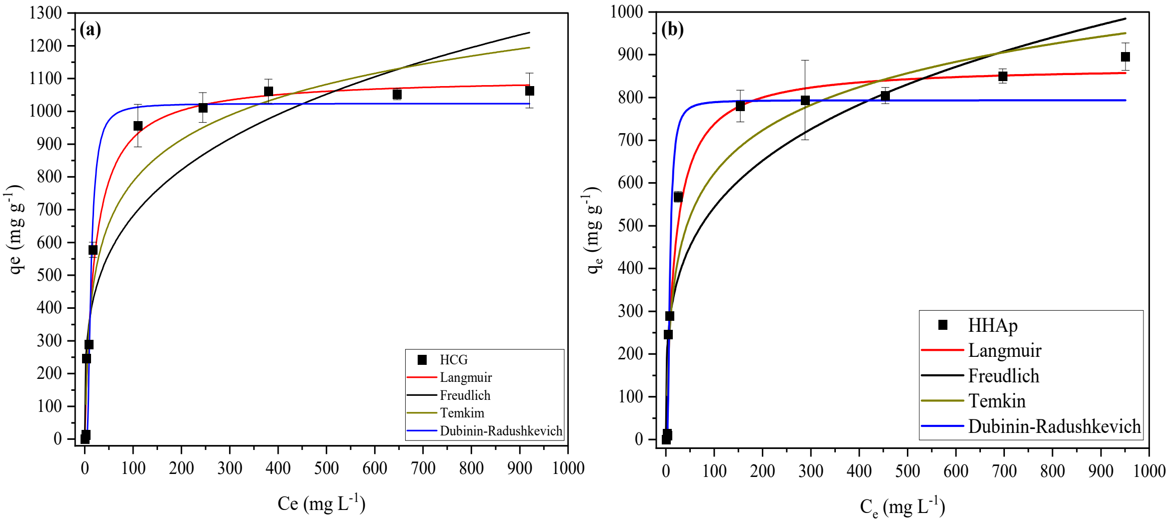

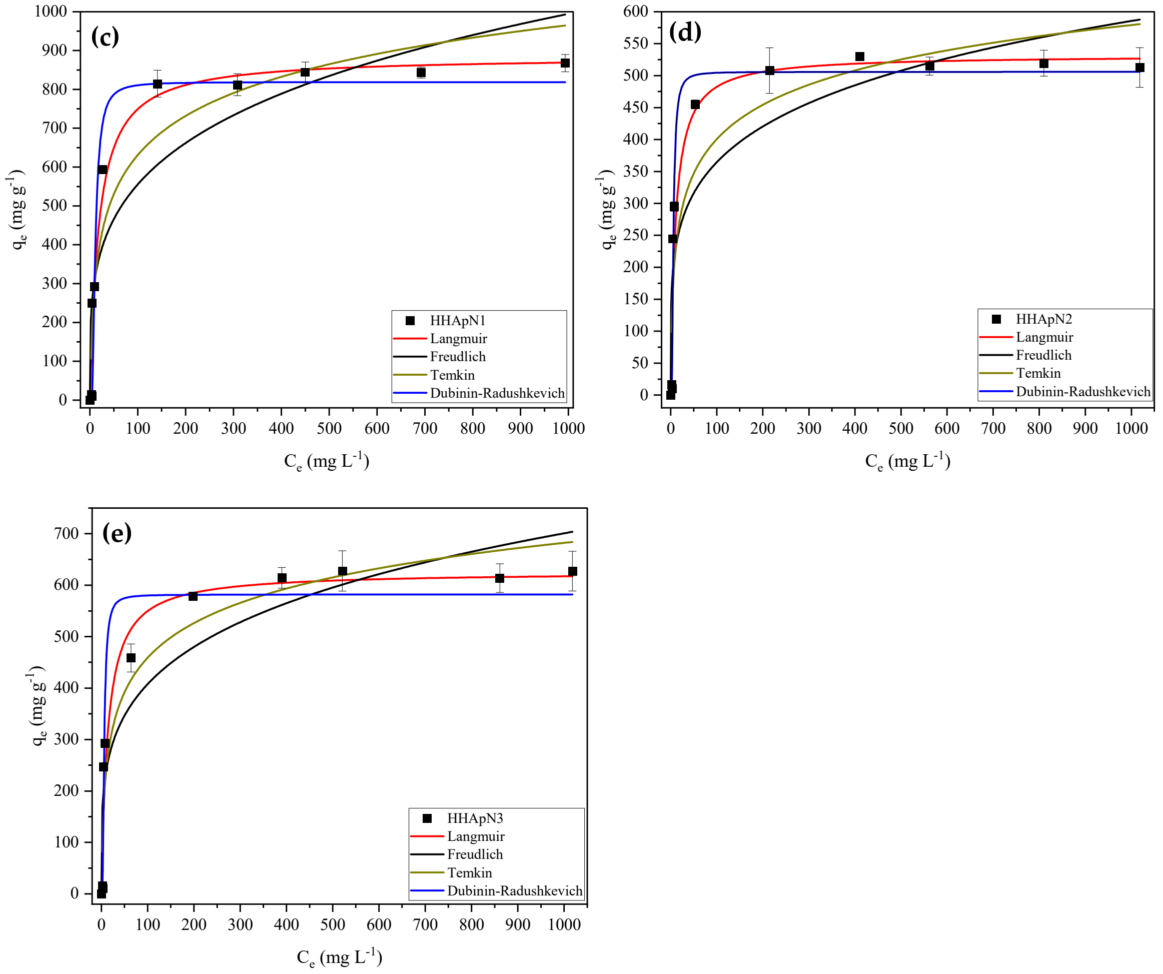

Figure S9. Equilibrium isotherms for methylene blue adsorption for nanocomposite hydrogels (a) HCG, (b) HHAp, (c) HHApN1, (d) HHApN2, and (e) HHApN3.

Table S5. Adsorption data of methylene blue dye by cashew gum nanocomposite aerogels obtained after fitting to the Langmuir, Freundlich, Temkin, and Dubinin-Raduskevich (D-R) models.

Model	Parameter	Sample				
		HCG	HHAp	HHApN1	HHApN2	HHApN3
	q_{max}(mg g^{-1})	1103.9	873.85	884.64	531.92	625.76
**Langmuir**	K_L(L mg^{-1})	0.0495	0.0541	0.0553	0,0973	0.0722
	R^2	0.980	0.968	0.970	0.939	0.951
	χ^2	4666.3	4854.4	4658.4	3413.6	3853.4
**Freundlich**	K_f(L g^{-1})	196.2	160.9	173.2	141.1	138.0
	R^2	0.874	0.875	0.854	0.815	0.872
	χ^2	30620.7	18929.9	22739.8	10422.5	10133.9
**Temkin**	A_t (L g^{-1})	0.712	0.714	0.769	1.748	1.138
	R^2	0.956	0.947	0.935	0.885	0.936
	χ^2	10568.2	7994.2	10029.0	6492.0	5083.2
**D-R**	q_{max}(mg g^{-1})	1023.8	793.53	818.89	505.87	581.74
	R^2	0.962	0.945	0.946	0.971	0.942
	χ^2	9190.3	8253.1	8398.2	1583.5	4593.6

(a)

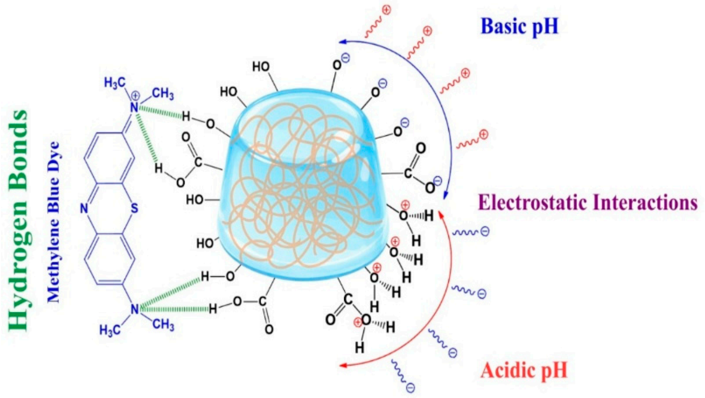

(b)

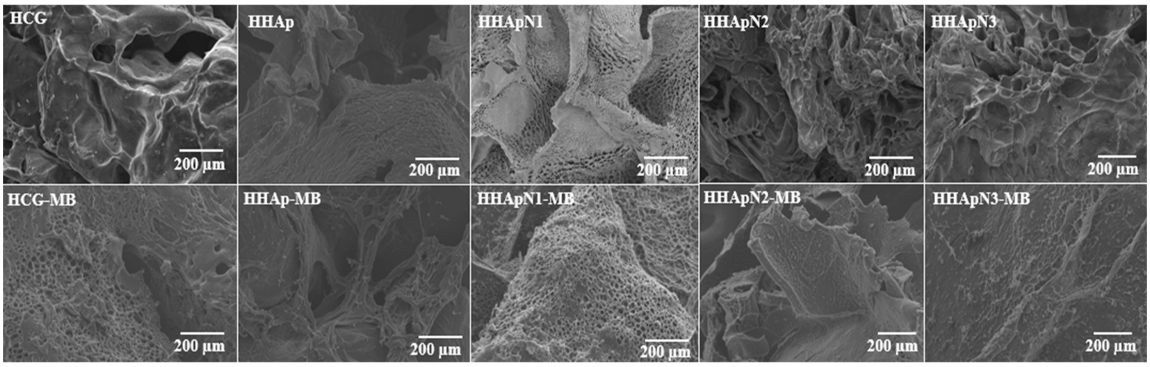

Figure S10. (a) Schematic representation of the proposed methylene blue adsorption mechanism by aerogels as a function of the pH of the medium; (b) SEM images of HCG, HHAp, HHApN1, HHApN2, and HHApN3 aerogels before (images above) and after adsorption (images below) of methylene blue.
